# Early Cretaceous troodontine troodontid (Dinosauria: Theropoda) from the Ohyamashimo Formation of Japan reveals the early evolution of Troodontinae

**DOI:** 10.1038/s41598-024-66815-2

**Published:** 2024-07-25

**Authors:** Katsuhiro Kubota, Yoshitsugu Kobayashi, Tadahiro Ikeda

**Affiliations:** 1 Museum of Nature and Human Activities, Hyogo, Sanda, Hyogo 669‑1546 Japan; 2 Institute of Natural and Environmental Sciences, University of Hyogo, Sanda, Hyogo 669‑1546 Japan; 3 Hokkaido University Museum, Hokkaido University, Sapporo, Hokkaido 060‑0810 Japan

**Keywords:** Arctometatarsus, Geometric morphometric analysis, Sleeping posture, Theropoda, Troodontidae, Troodontinae, Palaeontology, Palaeontology

## Abstract

A new troodontid dinosaur, *Hypnovenator matsubaraetoheorum* gen. et sp. nov., is described based on an articulated postcranial skeleton recovered from the fluvial deposits of the Albian Ohyamashimo Formation of the Sasayama Group in Tambasasayama City, Hyogo Prefecture, Japan. *Hypnovenator* is distinguished from other troodontids by four autapomorphies and a combination of additional features. Our phylogenetic analysis positions *Hypnovenator* as the oldest and one of the most basal troodontines, forming a clade with *Gobivenator mongoliensis*. The discovery of *Hypnovenator* suggests that small-bodied maniraptorans with a sleeping posture were common not only in environments with volcanic and eolian events or alluvial systems but also in fluvial systems. Geometric morphometric analysis of manual ungual phalanges shows that manual ungual phalanges I and III of *Hypnovenator* exhibit considerable morphological variation but are functionally similar, which differs from those of non-troodontine troodontids, reflecting the transition of manual motion within Troodontinae. *Hypnovenator* also has mosaic features in the pes related to cursoriality. This study reveals that asymmetrical arctometatarsus occurred by the Albian, and some morphological changes, such as shorter digit IV than digit III and non-ungual phalanges of digits III with roller joints and digit IV with weakly ginglymoid articulation, arose during the early Late Cretaceous.

## Introduction

Troodontidae is a clade of small-bodied and gracile theropod dinosaurs^1^. Although the phylogenetic position of Troodontidae is traditionally considered a clade with Dromaeosauridae, forming Deinonychosauria^1–9^, Troodontidae is also regarded as a sister clade to Avialae^10,11^. *Anchiornis* from the Late Jurassic of China is problematic in its phylogeny and is included in Troodontidae^2,4–7,11^ or Avialae^10,12,13^. These active discussions significantly improve our understanding of the phylogeny and osteology of non-avian theropod and greatly influence our comprehension of early avialan evolution^8,14^. Since the discovery of the first troodontid *Troodon* in the Upper Cretaceous of Canada^15^, troodontid materials have been discovered from the Middle Jurassic to Upper Cretaceous of Asia, Europe, and North America^1,9,16^. However, troodontid specimens with articulation are extremely rare. Although well-preserved and articulated basal troodontid specimens have been found in the Barremian deposits of China over the last 20 years^6,7,17–20^, diagnosed troodontids from the middle Cretaceous are represented by only two taxa, *Sinornithoides*^21^ and *Urbacodon*^22^. *Sinornithoides* from China comprises a nearly complete skeleton with a sleeping posture, whereas *Urbacodon* from Uzbekistan consists only of a dentary with some teeth. Two non-named troodontid specimens, MPC-D 100/44^23^ and 100/140^5^, are fragmentary. Recent phylogenetic studies have identified relatively stable clades such as Sinovenatorinae and Troodontinae. Jinfengopteryginae is another potential clade^16^ but has been unstable in other studies^10^.

**Figure 1:**
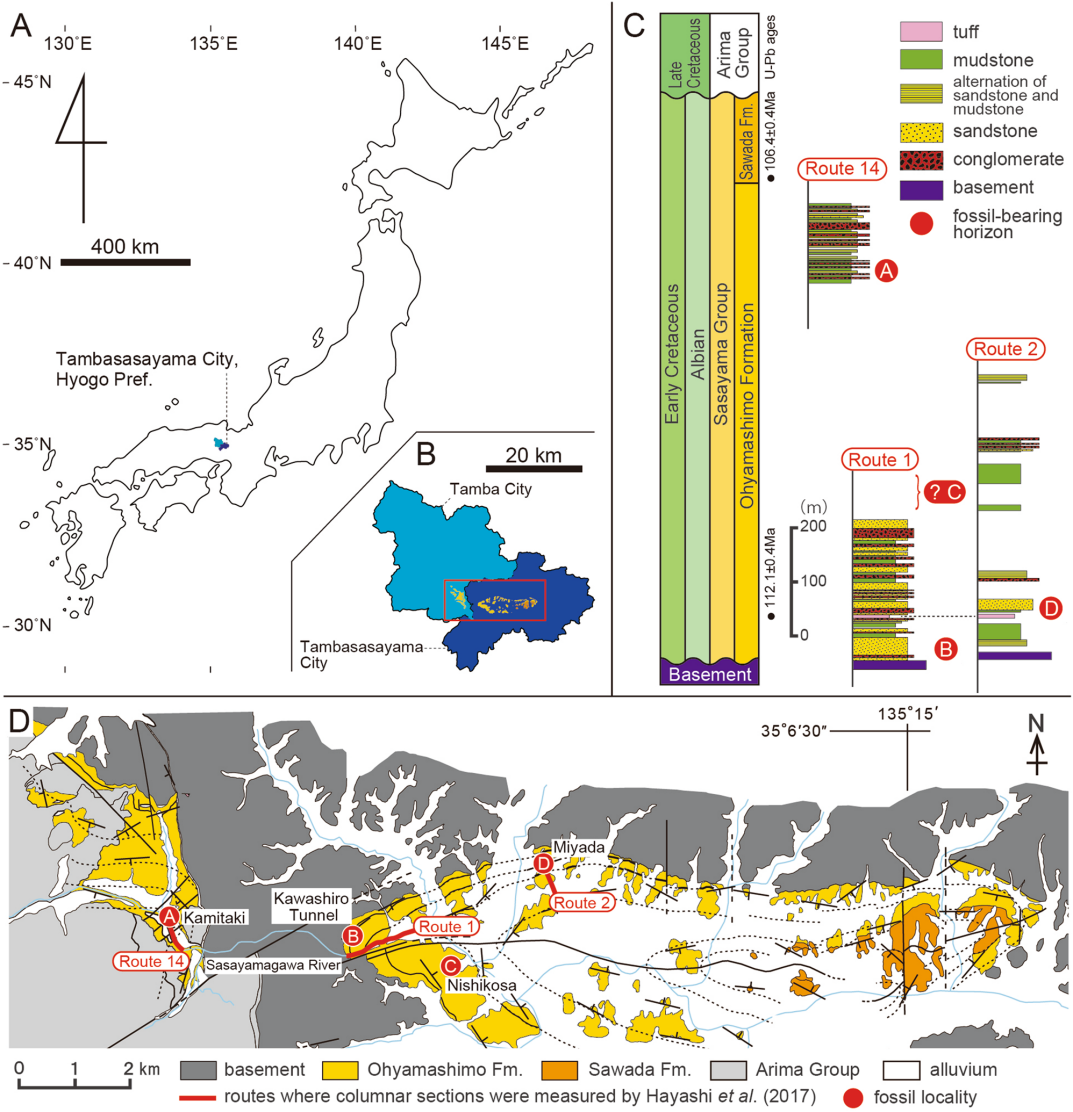
Locality maps and geology in Tambasasayama and Tamba cities, Hyogo Prefecture, Japan. (**A**) Map of Japan showing the locations of Tambasasayama (dark blue) and Tamba cities (light blue) in Hyogo Prefecture. (**B**) Distribution of the Sasayama Group in Tambasasayama and Tamba cities. (**C**) Stratigraphic sections of the Sasayama Group, adapted from Hayashi et al.^25^. U–Pb ages in the lower parts of the Ohyamashimo and Sawada formations were obtained by Kusuhashi et al.^26^. The routes for the stratigraphic sections are shown in (D). The stratigraphic positions of the localities (**A**) Kamitaki, (**B**) Kawashiro Tunnel, (**C**) Nishikosa (where *Hypnovenator matsubaraetoheorum* gen. et sp. nov. was recovered), and (**D**) Miyada are indicated by red circles. (**D**) Geological map of the Sasayama Group, fossil localities, and routes for stratigraphic sections. This map is after Yoshikawa^27^. This figure was created using Adobe Illustrator 28.3 (https://www.adobe.com/).

In September 2010, a partial theropod skeleton including the forelimb and knee was discovered in crushing rocks from the Ohyamashimo Formation during the construction of a public park at Nishikosa in Tambasasayama City, Hyogo Prefecture (Fig. 1). This discovery was made by Mrs. Kaoru Matsubara and Takaharu Ohe, members of an amateur group “Research Group on the Sasayama Group (Sasayama-sougun wo shiraberu kai)”. In July 2011, an articulated theropod heel was collected from the same locality during an excavation organized by the Museum of Nature and Human Activities, Hyogo. These specimens were identified as a troodontid theropod^24^, which is an only confirmed occurrence of a troodontid in Japan (Supplementary Text S1). Here we describe the Nishikosa material, test its phylogenetic position within troodontids, quantify the shapes of the ungual phalanx, and discuss the implications for the manual and pedal evolution of Troodontidae.

### Institutional abbreviations

MNHAH, Museum of Nature and Human Activities, Hyogo, Sanda, Hyogo, Japan; MPC, Institute of Paleontology, Mongolian Academy of Sciences, Ulaanbaatar, Mongolia; SDUST, Vertebrate Palaeontological Collection of College of Earth Science and Engineering, Shandong University of Science and Technology (Qingdao, China).

## Results

### Geological setting

The Ohyamashimo Formation, the lower unit of the Sasayama Group, in Tambasasayama and Tamba cities of Hyogo Prefecture (Fig. 1A,B), consists of sandstones, mudstones, and conglomerates, representing fluvial deposits under a semi-arid to subhumid climate^25^. This formation has yielded numerous dinosaur remains and eggshells^28^. Isolated teeth from some localities include tyrannosauroids, therizinosaurs, dromaeosaurids, sauropods, iguanodontians, and ankylosaurs^24,29^, while the skeletons of a sauropod (*Tambatitanis*) and neoceratopsian are found in Kamitaki^30^ and at three localities (Kawashiro Tunnel^31^, Miyada^32^, and Nishikosa^24^), respectively (Fig. 1D). Nishikosa (35° 04ʹ 30″ N, 135° 09ʹ 35″ E) is located at the western edge of the Sasayama Basin. Unfortunately, despite several excavations, the theropod-bearing horizon has not been relocated at Nishikosa.

Nishikosa is placed approximately 1 km southeast of the eastern end of Route 1 for the stratigraphic section (Fig. 1D), where the lower part of the Ohyamashimo Formation is exposed^25^. The beds around the localities show NW–SE at the strike and 30°NE at the dip^27^. Nishikosa sits about 20–60 m higher in altitude than the eastern end of Route 1, indicating that the theropod-bearing horizon at Nishikosa can be temporally correlated above the upper limit of the stratigraphic section in Route 1, likely within the middle part of the Ohyamashimo Formation (Fig. 1C). U–Pb ages of zircons indicate 112.1 ± 0.4 Ma for the lowermost part of the Ohyamashimo Formation and 106.4 ± 0.4 Ma for the lower part of the overlying Sawada Formation^26^. Thus, the middle part of the Ohyamashimo Formation is assigned to be the early to middle Albian in age.

## Systematic paleontology

Theropoda Marsh^33^.

Coelurosauria von Huene^34^.

Troodontidae Gilmore^35^.

*Hypnovenator matsubaraetoheorum* gen. et sp. nov.

### ZooBank ID

urn:lsid:zoobank.org:pub:BF77721B-211E-4190-B012-3669BD1221AA (for this publication), urn:lsid:zoobank.org:act:AF64F61F-8854-42E2-8F7C-6645242534CB (for the new genus), and urn:lsid:zoobank.org:act:C3398111-FCE7-4624-AE03-38170349345D (for the new species).

### Etymology

The genus name derives from “*hypno*”, refers to “sleep” in ancient Greek, and “*venator*”, means “hunter” in Latin. The specific name, “*matsubaraetoheorum*”, honors Mrs. Kaoru Matsubara and Takaharu Ohe, who are the first discoverers of a block including a part of *Hypnovenator matsubaraetoheorum* holotype specimen.

### Holotype

**Figure 2:**
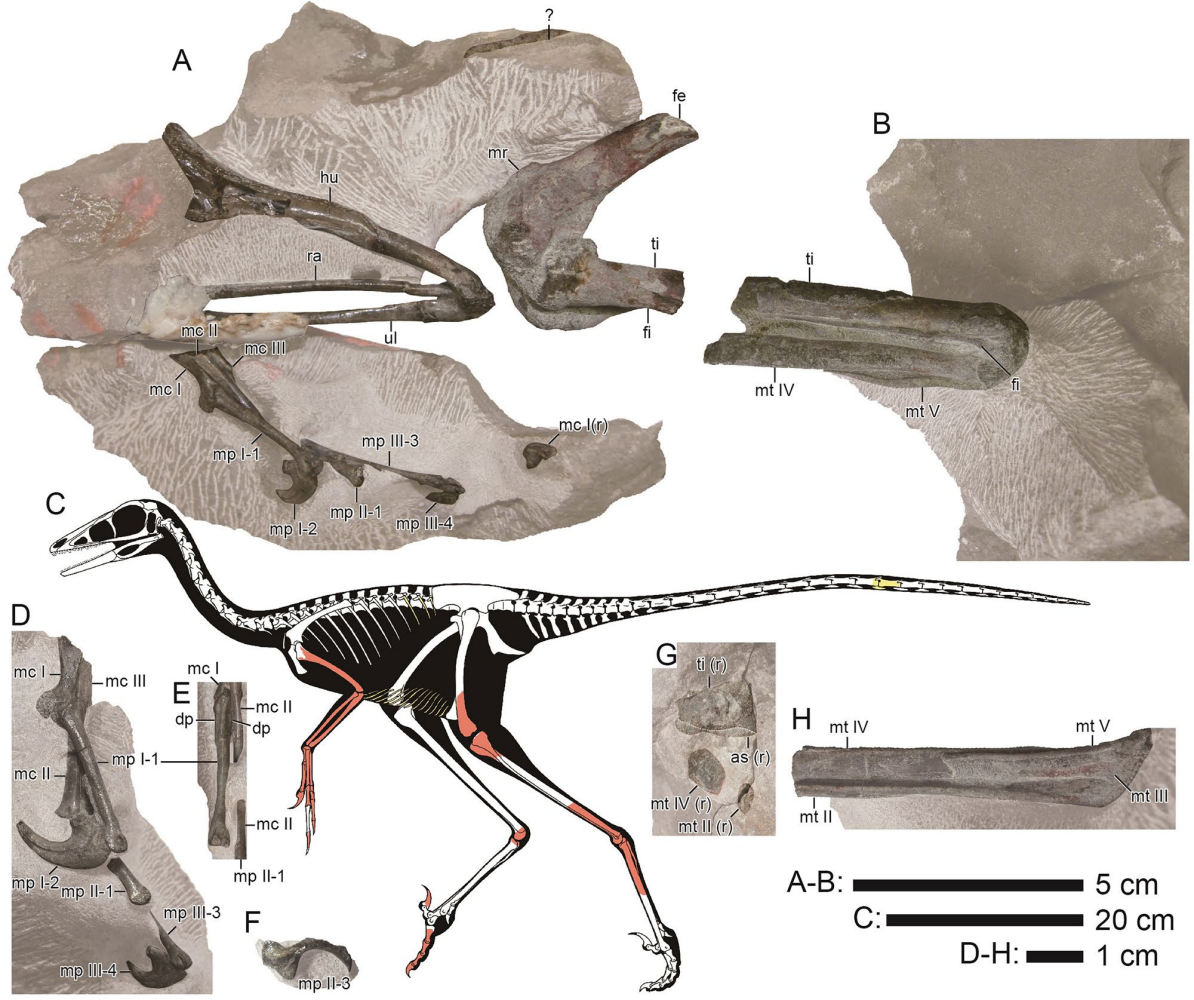
*Hypnovenator matsubaraetoheorum* gen. et sp. nov. Blocks including the forelimb, knee (**A**), and heel (**B**). (**C**) Reconstructed skeleton. Red and yellow colors show the confirmed and probable positions of the recovered elements, respectively (Courtesy of Genya Masukawa). (**D**) Left manus in medial view. (**E**) Left manual phalanx I-1 in dorsal view. (**F**) Removed fragmentary left manual phalanx II-3 (manual ungual phalanx II) for preparing the left manus. (**G**) Cross-section of the bent right ankle. (**H**) Left metatarsus in posterior view. Abbreviations: *as*, astragalus; *dp*, depression; *fe*, femur; *fi*, fibula; *hu*, humerus; *mc* *I*, metacarpal I; *mc II*, metacarpal II; *mc III*, metacarpal III; *mp I-1*, manual phalanx I-1; *mp I-2*, manual phalanx I-2 (manual ungual phalanx I); *mp II-1*, manual phalanx II-1; *mp II-3*, manual phalanx II-3 (manual ungual phalanx II); *mp III-3*, manual phalanx III-3; *mp III-4,* manual phalanx III-4 (manual ungual phalanx III); *mr*, medial ridge; *mt II*, metatarsal II; *mt III*, metatarsal III; *mt IV*, metatarsal IV; *mt* *V*, metatarsal V; *ra*, radius; *ti*, tibia; *ul*, ulna. Almost all elements are from the left side. Abbreviations for elements from the right side added ‘(r)’ at the end. This figure was created using Adobe Photoshop 25.5.1 and Adobe Illustrator 28.3 (https://www.adobe.com/).

**Figure 3:**
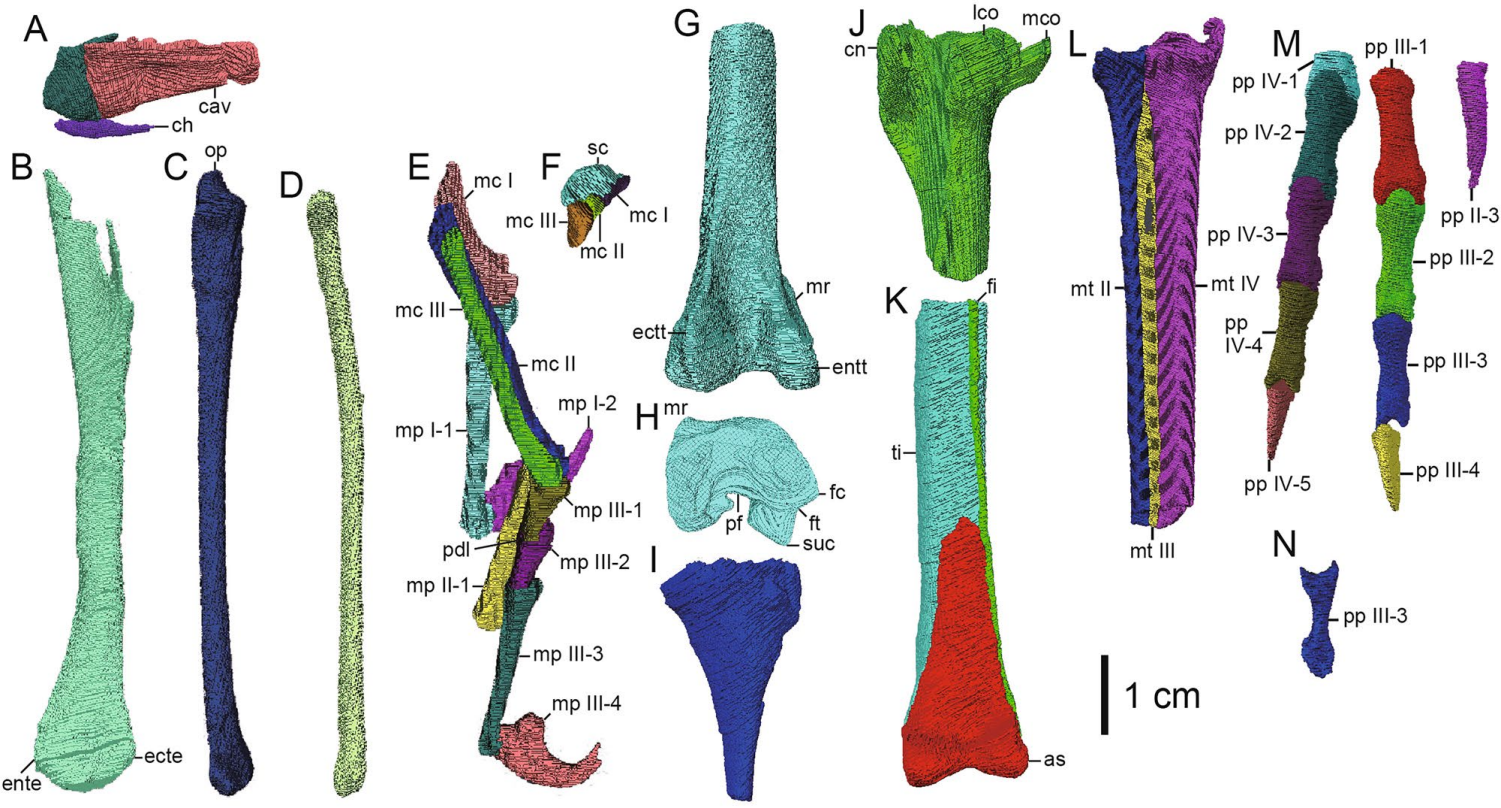
Selected elements of *Hypnovenator matsubaraetoheorum* gen. et sp. nov. (**A**) Distal caudal vertebrae and a chevron in left lateral view. (**B**) Left humerus in anterior view. (**C**) Left ulna in medial view. (**D**) Left radius in medial view. (**E**) Left manus, missing the proximal ends of metacarpals I-III and manual phalanges II-2 to 3, in lateral view. (**F**) Left distal carpal and proximal ends of metacarpals I-III in ventral view. Distal part of left femur in posterior (**G**) and distal (**H**) views. (**I**) Proximal part of left fibula in lateral view. (**J**) Proximal part of left tibia in lateral view. (**K**) Distal parts of tibia and fibula, astragalus, and calcaneum from the left side in anterior view. (**L**) Proximal half of left metatarsals II-IV in anterior view. (**M**) Right pedal phalanges II-3, III-1 to 4, and IV-1 to 5 in dorsal view. (**N**) Right pedal phalanx III-3 in lateral view. Abbreviations: *as*, astragalus; *cav*, caudal vertebra; *ch*, chevron; *cn*, cnemial crest; *ecte*, ectepicondyle; *ectt*, ectocondylar tubercle; *ente*, entepicondyle; *entt*, entocondylar tubercle; *fc*, fibular crest; *fi*, fibula; *ft*, fibular trochlea (= trochlea fibularis); *lco*, lateral condyle; *mc I*, metacarpal I; *mc II*, metacarpal II; *mc III*, metacarpal III; *mco*, medial condyle; *mp I-1*, manual phalanx I-1; *mp I-2*, manual phalanx I-2 (manual ungual phalanx I); *mp II-1*, manual phalanx II-1; *mp III-1*, manual phalanx III-1; *mp III-2*, manual phalanx III-2; *mp III-3*, manual phalanx III-3; *mp III-4*, manual phalanx III-4 (manual ungual phalanx III); *mr*, medial ridge; *mt II*, metatarsal II; *mt III*, metatarsal III; *mt IV*, metatarsal IV; *op*, olecranon process; *pdl*, proximodorsal lip; *pf*, popliteal fossa; *pp II-3*, pedal phalanx II-3 (pedal ungual phalanx II); *pp III-1*, pedal phalanx III-1; *pp III-2*, pedal phalanx III-2; *pp III-3*, pedal phalanx III-3; *pp III-4*, pedal phalanx III-4 (pedal ungual phalanx III); *pp IV-1*, pedal phalanx IV-1; *pp IV-2*, pedal phalanx IV-2; *pp IV-3*, pedal phalanx IV-3; *pp IV-4*, pedal phalanx IV-4; *pp IV-5*, pedal phalanx IV-5 (pedal ungual phalanx IV); *sc*, semilunate carpal; *suc*, supracondylar crest (= lateral posterior ridge, tibiofibular crest); *ti*, tibia. This figure was created using Adobe Photoshop 25.5.1 and Adobe Illustrator 28.3 (https://www.adobe.com/).

MNHAH D1033340, consisting of two caudal vertebrae, two dorsal ribs, thirty-eight gastralia, a chevron, left humerus, left radius, left ulna, left carpal, left metacarpals I to III, the distal end of right metacarpal I, left manual phalanges I-1, I-2, II-1, II-3, and III-1 to 4, the distal part of left femur, left tibia and fibula missing the mid-shaft, the distal end of right tibia, left astragalus, the distal end of right astragalus, the proximal parts of left metatarsals II to V, the proximal ends of right metatarsals II and IV, right pedal phalanges II-3, III-1 to 4, and IV-1 to 5 (Figs. 2, 3; Supplementary Figs. S1–S13 and Table S1).

### Locality and horizon

Hyogo Prefectural Tamba Namikimichi Central Park at Nishikosa, Tambasasayama City, Hyogo Prefecture, Japan; the early to middle Albian (112.1–106.4 Ma^26^) Ohyamashimo Formation of the Sasayama Group.

### Diagnosis

A troodontid with the following unique characters: a pair of proximodistally extended depressions on the proximodorsal surface of manual phalanx I-1; long dorsal and ventral proximal lips on manual phalanx III-2 for tight articulation with phalanx III-1; a proximodistally longitudinal medial ridge on the anterior surface of the femur proximal to the medial condyle; and distorted distal condyles with a widely convex distoventral margin on pedal phalanx III-3. Additionally, it is characterized by the following combination of two features: the thickest portion near the middle portion of the distal end of the ulna, and an angle of less than 11 degrees between the anterior edge of the cnemial crest and the anterior edge of the tibial shaft.

### Description

Theropod bones found in 2010 (Fig. 2A) and 2011 (Fig. 2B) were assigned to a single individual skeleton (Supplementary Text S2). The left articulated forelimb bones are folded at angles of 26 degrees at the elbow and 53 degrees at the wrist. Medial to the forelimb, the disarticulated gastralia are arranged in subparallel and form angles of 100 to 130 degrees with the long axis of the humerus. Ventral to the posterior half of the gastralia area, right pedal digits extend without any fold. Medial to the right pedal digits, the left articulated femur and tibia are folded at an angle of 42 degrees. Both ankles are tightly folded. If the posterior surface of the left tibia is considered as a horizontal plane, the right ankle is positioned 40 mm anterior and 50 mm dorsal to the left one. The tail bones are just medial to the left ankle and nearly vertical to the long axis of the left tibia.

The preserved length of the caudal centrum is three times as long as the height of the anterior articular surface (Fig. 3A; Supplementary Fig. S1). The lateral surfaces have a shallow concavity along the central length, showing that the midpoint of the centrum is hourglass-shaped in cross-section, as in *Daliansaurus*, *Gobivenator*, *Sinornithoides*, and MPC-D 100/140^5,7,21,36^, but unlike *Troodon*, *Urbacodon*, and *Zanabazar*, which have smooth lateral surfaces^37–39^. Based on the remarkedly long centrum without transverse processes, the caudal vertebra may be assigned to one posterior to 9th^4,6,7,36^. The distal chevron is dorsoventrally compressed and anteroposteriorly longer than high (Fig. 3A).

The preserved shafts of the dorsal rib are lateromedially wide and have flat or slightly convex anterior and posterior surfaces (Supplementary Fig. S2), resembling the mid-shaft of the posterior dorsal rib in *Liaoningvenator* and *Troodon*^6,40^ rather than those of the anterior to middle ones in the taxa with lateromedially narrow rib shafts. In at least three of thirty-eight rod-like gastralia, a half of the shaft is straight with an expanded end, whereas the rest is curved with a thinner end. This morphology is consistent with the medial segments^17,21,40–42^. Three other segments are longer and slenderer than the medial ones and are assigned as the lateral segments^19,21^.

The humeral shaft is posteriorly bowed as in *Gobivenator* and *Liaoningvenator*^6,36^, but unlike *Daliansaurus*, *Jianianhualong*, *Linhevenator*, and *Mei*, which have a straight humeral shaft^4,7,11,20^ (Figs. 2A, 3B; Supplementary Fig. S3). The expanded distal end is 231% as wide as the mid-shaft and close to 257% in *Gobivenator* (MPC-D 100/86). The ulna is almost straight with a slightly bowed distal extent unlike *Daliansaurus*, *Sinornithoides*, and *Talos*, which have a slightly bowed shaft^3,7,21^, and *Jianianhualong* and *Mei*, which bear a strongly bowed shaft^4,20^ (Figs. 2A, 3C; Supplementary Fig. S4). The mid-shaft of the ulna is 71% as dorsoventrally high as that of the radius, resembling *Sinornithoides* (69%)^21^ and *Daliansaurus* (74%)^7^. The subtriangular proximal end has a single concave articular facet as in *Gobivenator* (MPC-D 100/86). The distal end is 148% wider than its maximum height, which is positioned in the middle portion. The radius is slightly curved dorsally as in *Jianianhualong* and *Xiaotingia*^20,43^ and unlike *Mei* and *Sinornithoides*, which have a straight shaft^4,21^ (Figs. 2A, 3D; Supplementary Fig. S5). The semilunate carpal has a transverse trochlear groove on the proximal surface, a mediodorsal process for articulation with metacarpal I, and a ventrolateral process for covering the ventral side of the proximal end of metacarpal III (Fig. 3F; Supplementary Fig. S6). A small distal carpal 3 is found in some basal troodontids^44^ but not identified in *Hypnovenator* because the carpal fuses with the semilunate carpal to form the ventrolateral process. Metacarpal I has a concave medial edge, forming a ventromedial flange (Figs. 2A,D–E, 3E,F; Supplementary Figs. S6, S7). The distal articular facet is deeply ginglymoid by an intercondylar groove with a higher lateral condyle than the medial one. The orientation of the groove is nearly parallel to that of metacarpal II. Metacarpal II is straight and nearly parallel to metacarpal III. The distal articular facet is ginglymoid with a higher medial condyle than the lateral one. Metacarpal III is straight as in *Sinornithoides* and MPC-D 100/44^21,23^ and unlike *Daliansaurus* and *Mei*, which have a moderately curved shaft^4,7^. The distal articular facet is rounded. All non-ungual manual phalanges are narrow and elongate with a distal ginglymoid condyle with the exception of phalanges III-1 and III-2 (Figs. 2A,D,F, 3E; Supplementary Fig. S7). The dorsal surface of the proximal phalanx I-1 possesses a pair of subtriangular and shallow fossae, which are dorsally divided by a faint longitudinal ridge. The fossae are 8.2 mm long and 2.1 mm high on the lateral side and 9.1 mm long and 2.2 mm high on the medial side. Phalanx I-1 bears a prominent proximoventral heel, twice as wide as the mid-shaft, as in phalanx II-1. Ungual phalanx I is highly curved and bears a proximally placed and prominent flexor tubercle, which is slightly lower than the articular facet. Dorsal to the facet, ungual phalanx I lacks a proximodorsal lip as in *Sinornithoides* and *Xixiasaurus*^21,45^. Phalanx II-1 is similar to phalanx I-1 in morphology except for lacking a pair of fossae in the proximodorsal surface as seen in phalanx I-1. Ungual phalanx II has a highly curved ventral edge and a proximally placed flexor tubercle. However, it is difficult to further compare other ungual phalanges due to its fragmentary condition. The proximal articular facet of phalanx III-1 is not transversely expanded, which differs from phalanges I-1 and II-1, but has a proximoventral heel with 185% as high as the minimum height of the shaft just proximal to the distal condyles. The proximal articular facet of phalanx III-2 bears long dorsal and ventral lips for tight articulation with phalanx III-1. This immovable articulation between phalanges III-1 and III-2 is also known in SDUST-V1042 lacking the proximodorsal lip of phalanx III-2^46^. Phalanx III-3 is barely flexed because its proximoventral lip contacts the ventrodistal surface of phalanx III-2 with slight flexion. Ungual phalanx III is shorter than ungual phalanx I, and the dorsal arch of ungual phalanx III is higher than the level of the dorsal extremity of the proximal articular facet with the proximal articular surface of ungual phalanx orientated vertically. The highest point of the dorsal arch is positioned at a half of the ungual phalanx length, which is more distal than those of other troodontids^5,20,21,43^. A part of the keratinous sheath is preserved in contact with the distal tip.

The distal femur bears a shallow notch to separate the supracondylar crest from the lateral condyle as in *Gobivenator*^36^ but unlike *Almas* and *Daliansaurus*^7,41^, which have a smooth distal edge of the crest (Figs. 2A, 3G–H; Supplementary Fig. S8). A thick and longitudinal ridge with 15.6 mm in length and 3.6 mm in height extends proximomedially in the anteromedial edge of the distal femur. The position of this ridge resembles a prominent process in *Linhevenator* and *Philovenator*, but which is low mound-like^11,47^. If the ridge is homologous to the process in the two taxa, the ridge is remarkedly developed from the process. In lateral view, the straight anterior edge of the tibial cnemial crest is nearly parallel to the tibial shaft unlike *Almas*^41^, *Gobivenator* (MPC-D 100/86), *Liaoningvenator*^6^, and *Sinusonasus*^18^, which have an inclined anterior edge of the crest (Figs. 2A,B,G, 3J–K; Supplementary Figs. S9, S11). The medial surface of the proximal fibula bears a shallow concavity unlike *Troodon* with a pronounced fossa^3^ and *Mei*, *Talos*, and *Xiaotingia* with a flat medial surface^3,4,43^ (Figs. 2A,B, 3I,K; Supplementary Figs. S10, S11). The astragalus is fused with the calcaneum unlike *Talos* having no fusion^3^ (Figs. 2G, 3K; Supplementary Fig. S11). The distal condyles are anteroventrally divided by a shallow intercondylar groove resembling *Gobivenator* and *Mei* but unlike *Borogovia*, *Talos*, and *Troodon*, which possess a deeper intercondylar groove^3,4,10,36,40^. The ascending process is triangular with its center along the midline, resembling the general shape in *Gobivenator* and *Philovenator*^36,47^, unlike *Talos* and *Troodon* with a medially displaced process^3^. The base of the process bears a shallow depression as in *Talos*, *Troodon*, and *Zanabazar*^3,38^, but unlike *Liaoningvenator* and *Philovenator*, which have a flat anterior surface^6,47^. The ratio of metatarsals IV to II at the mid-shaft in posterior view is 250%, which is higher than those of most troodontids except for *Gobivenator* (272%)^36^ and *Talos* (276%)^3^ (Figs. 2B,G–H, 3L; Supplementary Fig. S12). Metatarsal III is strongly pinched in a trough formed between metatarsals II and IV along its shaft. Anteriorly, the proximal end of metatarsal III is obscured by the anteroproximal contact between metatarsals II and IV, forming an arctometatarsalian condition^48^. The anterior exposure of the proximal metatarsal III is wider until one fifth of the preserved length from distal to the proximal articular surface and narrower distally unlike *Linhevenator* with a wider anterior exposure distally^11^ and *Philovenator* with no anterior exposure in the proximal extent^47^. All non-ungual pedal phalanges are slender with a width of less than 30% of their length and have a ginglymoid distal articular surface (Fig. 3M,N; Supplementary Fig. S13). Ungual phalanx II has a mediolaterally compressed elliptical cross-section with a rounded ventral edge. Phalanx III-3 is the most dorsoventrally compressed phalanx of digit III, measuring 64% as high as its width at mid-shaft, compared with 90% in phalanges III-1 and III-2. Unlike other non-ungual phalanges, the distal condyles of phalanx III-3 bear a widely convex distoventral margin. The lateral distal condyle of phalanx III-3 extends distally. Ungual phalanx III is triangular in cross-section and has a small proximodorsal lip as in *Talos*^3^. Ungual phalanx IV is less curved than ungual phalanx III.

## Discussions

### Phylogenetic analysis

**Figure 4:**
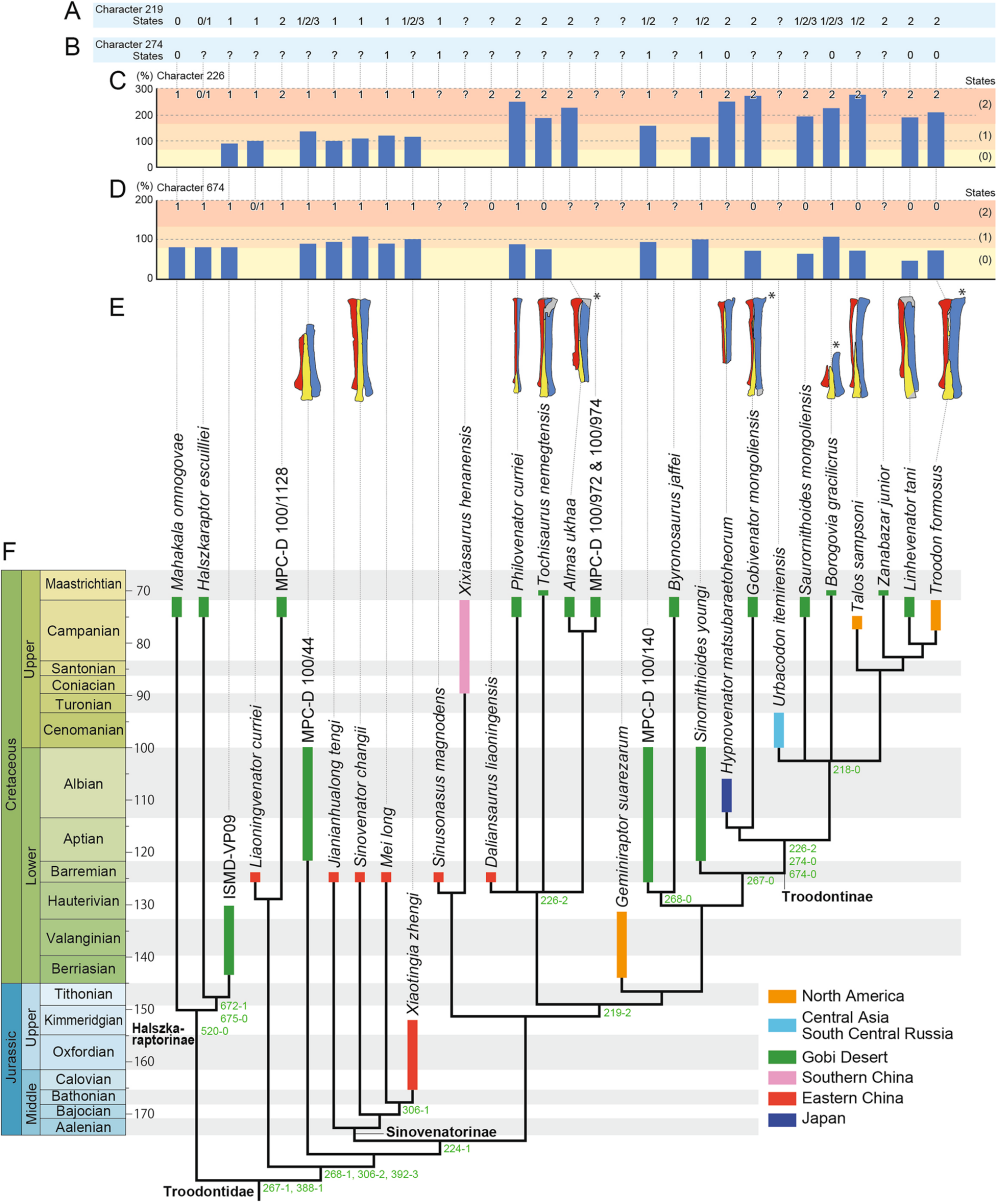
Phylogenetic relationships of *Hypnovenator matsubaraetoheorum* gen. et sp. nov. and states of four characters. (**A**) States of character 219 (proximal transverse constriction of metatarsal III: anterior exposure wider or subequal to metatarsals II and IV [0], subarctometatarsal [1], arctometatarsal [2], hyperarctometatarsal [3]). (**B**) States of character 274 (cross section of pedal ungual phalanges III and IV: expanded ventrally, triangular [0], subequal in transverse width dorsally and ventrally [1]). (**C**) Ratios for states of character 226 (ratio of metatarsals IV to II in transverse widths at midshaft in posterior view: < 66% [0], 66–165% [1], > 166% [2]). (**D**) Ratios for states of character 674 (ratio of metatarsals II to IV in transverse width of trochlea: < 80% [0], 80–130% [1],  > 130% [2]). (**E**) Comparison of troodontid metatarsus in anterior view. Red, yellow, and blue colors represent metatarsals II, III, and IV, respectively. Metatarsus with an asterisk is reserved from the original image. (**F**) Phylogenetic relationships of *Hypnovenator matsubaraetoheorum* gen. et sp. nov. Strict consensus phylogenetic tree (CI: 0.072, RI: 0.618) with character distribution of 10 most parsimonious trees of 12,235 steps. Green numbers on the right side of each branch show synapomorphies on the pes. For character descriptions, see Sellés et al.^16^. This figure was created using Adobe Illustrator 28.3 (https://www.adobe.com/).

The phylogenetic analysis produced 10 most parsimonious trees with 12,235 steps with a consistency index of 0.072 and a retention index of 0.618. A strict consensus tree revealed that *Hypnovenator* belongs to Troodontidae, having two of seven synapomorphies of the clade (minimum transverse width of metatarsus distally compared to the proximal width < 60% [character 388] and the posterior projection of the posterior surface just proximal to the lateral condyle of femur distinctly more posteriorly projected than the medial surface [character 657]) (Fig. 4; Supplementary Data S1 and Figs. S14, S15). Furthermore, the taxon is placed within Troodontinae, supported by two of eleven synapomorphies of the clade (transverse width at midshaft of metatarsal IV compared to metatarsal II > 166% [character 226] and expanded ventrally, triangular cross-section of pedal ungual phalanges III and IV [character 274]).

In addition to *Hypnovenator*, this study includes eight taxa (*Borogovia*, *Gobivenator*, *Linhevenator*, *Saurornithoides*, *Talos*, *Troodon*, *Urbacodon*, and *Zanabazar*) from the Upper Cretaceous as members of Troodontinae, which differs from previous works. Van der Reest and Currie^37^ placed *Latenivenatrix* in Troodontinae and excluded *Talos* from the clade, considering the former a junior synonym of *Stenonychosaurus*^49^, assigned to *Troodon* in this study^50^. Cau and Madzia^10^ included *Albertavenator*, *Almas*, *Philovenator*, and *Xixiasaurus* in Troodontinae and positioned *Borogovia* as a sister taxon to the clade. However, *Albertavenator* was not included in our analysis, and *Almas*, *Philovenator*, and *Xixiasaurus* were placed outside of Troodontinae. Two Early Cretaceous troodontids, *Geminiraptor* and *Sinornithoides*, were previously assigned within Troodontinae by Hartman et al.^8^ and Sellés et al.^16^, respectively, but our analysis placed them outside of the clade. With the exclusion of the two taxa from Troodontinae, *Hypnovenator* stands as the only Early Cretaceous troodontine troodontid, representing the oldest record of the clade.

*Hypnovenator* forms a clade with *Gobivenator*, positioned at the basal position of Troodontinae and united by four synapomorphies: a single proximal cotyla of the ulna [character 152], mediolateral width of the ascending process of astragalus < 58% width of astragalocalcaneum, when measured halfway up [character 209], flat anterior surface at base of the ascending process of astragalus compared to rest of its process [character 212], and 51–71% with a ratio of anteroposterior diameters at midshaft of radius to ulna [character 256].

### Posture

The partial skeleton of *Hypnovenator* was buried in muddy sandstone of fluvial deposits under low-energy water flow, containing plant fragments. It shows slight displacement but remain in proximity to their original positions, such as subparallel arranged gastralia segments, a tail positioned vertically to the long axis of the left tibia, and a right hindlimb shifted anterodorsally compared to the left one. *Hypnovenator* exhibits an intriguing posture, characterized by a loosely folded forelimb lateral to the gastralia, tightly folded ankles, and unbent pedal digits positioned under the gastralia, resembling the sleeping style of two Chinese non-troodontine troodontids (*Mei* and *Sinornithoides*)^17,51^. It has been suggested that the posture of the Chinese troodontids from the Lower Cretaceous may have indicated sheltering within a burrow or protective responses to volcanic and eolian events^4^. In contrast, two Mongolian alvarezsaurids (*Jaculinykus* and *Shuvuuia*) with a similar sleeping style^52,53^ have been reported from the Upper Cretaceous alluvial deposits^54,55^. This new report on a sleeping posture suggests that this posture was common within the clade of Troodontidae. Furthermore, it indicates that the sleeping posture of small-bodied maniraptorans is prevalent not only in environments with volcanic and eolian events or alluvial systems but also in fluvial systems.

### Evolution of manual ungual phalanges

**Figure 5:**
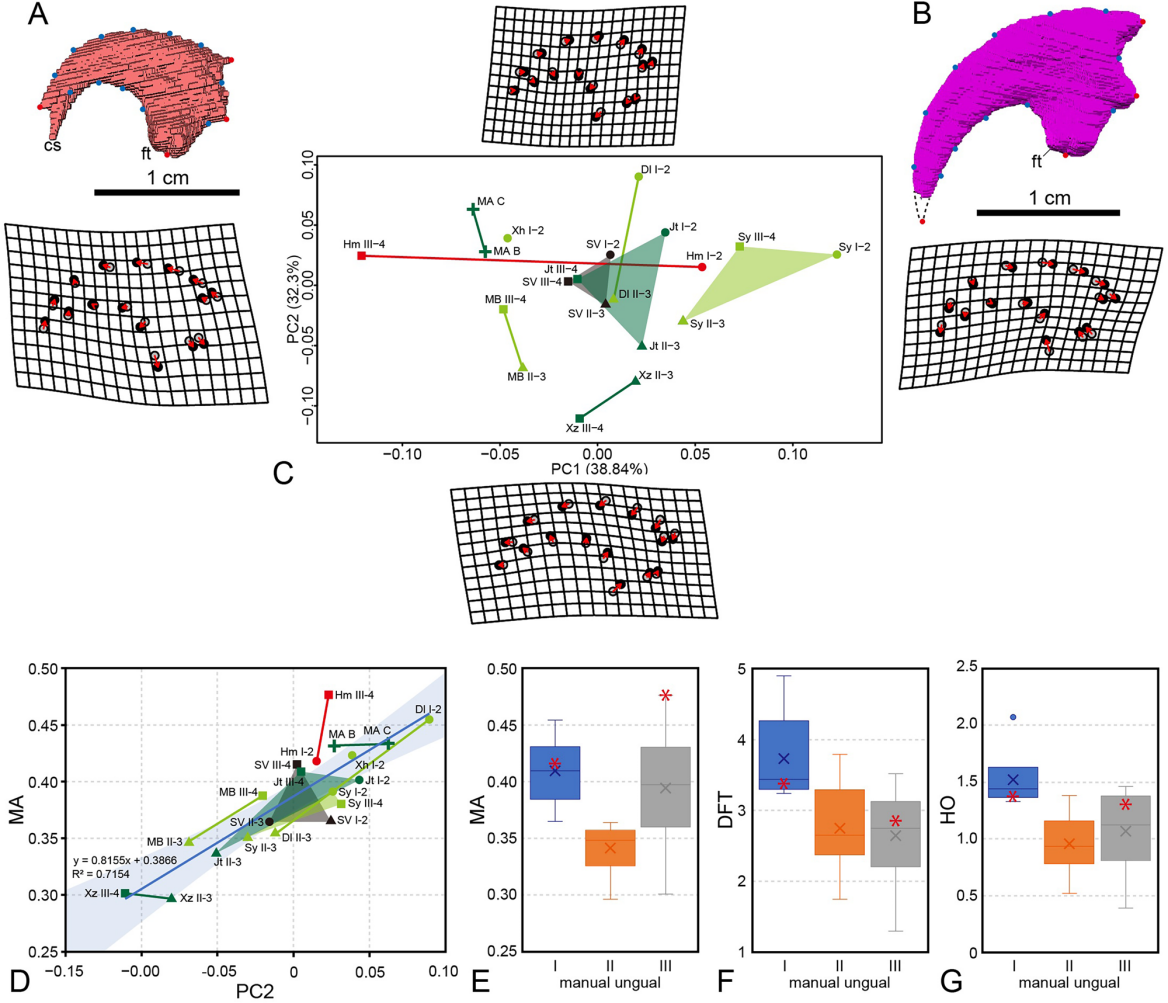
Manual ungual phalanges I (**A**) and III (**B**) of *Hypnovenator matsubaraetoheorum* gen. et sp. nov. Fixed and sliding landmarks are displayed on each ungual phalanx. Points are colored as follows: fixed landmarks (red) and sliding landmarks (blue). (**C**) Scatter plot showing PC1 and PC2 values obtained from geometric morphometric analysis. Symbols of manual ungual phalanges I-2, II-3, III-4, and unnumbered manual ungual phalanges on the graph are shown by circles, triangles, squares, and pluses, respectively. Dark green, light green, red, and black symbols are shown by members of Sinovenatorinae and the earlier branching clade than sinovenatorines, members of a sister clade to the Sinovenatorinae excluding *Hypnovenator matsubaraetoheorum*, *Hypnovenator matsubaraetoheorum* (= Troodontinae), and members with undecided position, respectively. The gray dots on the thin plate spline represent the “average” positions of the landmarks and the black dots represent the positions after deformation. (**D**) Regression plot of PC2 values obtained from geometric morphometric analysis and the mechanical advantage of troodontid ungual phalanges. The light blue area shows 95% confidence intervals. Boxplots represent the (**E**) mechanical advantages, (**F**) development of flexor tubercle, and (**G**) hypothesized output force in each ungual phalanx of troodontids. The red asterisk shows *Hypnovenator matsubaraetoheorum* in boxplots. Abbreviations: Dl, *Daliansaurus liaoningensis*; Hm, *Hypnovenator matsubaraetoheorum* ; Jt, *Jianianhualong tengi*; MA, MPC-D 100/44; MB, MPC-D 100/140; SV, SDUST V1042 (not included in our phylogenetic analysis); Sy, *Sinornithoides youngi*; Xh, *Xixiasaurus henanensis*; Xz, *Xiaotingia zhengi*; *cs*, claw sheath; *ft*, flexor tubercle; *DFT*, development of flexor tubercle; *MA*, mechanical advantage; *HO*, hypothesized output force. This figure was created using Adobe Photoshop 25.5.1 and Adobe Illustrator 28.3 (https://www.adobe.com/).

Geometric morphometric analysis of troodontid manual ungual phalanges revealed that more than 71% of the total shape variation is described by the first two principal components (PC1 and PC2) (Fig. 5C; Supplementary Data S6). PC1 is primarily associated with the curvature of ungual phalanges. This indicates that strongly curved ungual phalanges, with the highest point of the dorsal edge placed in the proximodorsal corner, are positioned on the positive side of PC1. While non-troodontine troodontids have PC1 values of higher than -0.07, *Hypnovenator* has the lowest PC1 value in ungual phalanx III (-0.12) and the third highest PC1 value in ungual phalanx I (0.05). This results in a remarkably wider PC1 range of 0.17 for the two ungual phalanges compared to non-troodontine troodontids, which generally have values of less than 0.08. The wide range in PC1 values may indicate a characteristic in Troodontinae. On the other hand, PC2 is related to the length and height of ungual phalanges and the size of the flexor tubercles. Short and high ungual phalanges with enlarged flexor tubercles are positioned on the positive side of PC2. *Hypnovenator* has nearly equal PC2 values in ungual phalanges I and III (0.02), similar to those in ungual phalanges I and III of *Sinornithoides* (0.03). With the exception of *Sinornithoides* and *Xiaotingia*, ungual phalanx II shows a positive shift in PC1 and a negative shift in PC2 from ungual phalanx III, whereas ungual phalanx III demonstrates negative shifts in both PC1 and PC2 from ungual phalanx I. The mechanical advantage (MA) of ungual phalanges shows a stronger correlation with PC2 than with PC1 (R^2^ = 0.7154, p < 0.0001, n = 20) (Fig. 5D). The MA value of ungual phalanx III of *Hypnovenator* is the highest (0.48), resulting in the ungual phalanx being exceptionally high from the regression line and plotted well outside of 95% confidence intervals. Boxplots of MAs in each ungual phalanx show that both ungual phalanges I and III are higher than ungual phalanx II, except for ungual phalanx III of *Xiaotingia*, which has a notably low MA value (0.30), and that ungual phalanx I of *Hypnovenator* is plotted near the median value of MA (Fig. 5E). Among troodontids, ungual phalanx I tends to have a larger flexor tubercle than other ungual phalanges (Fig. 5F). Both preserved ungual phalanges of *Hypnovenator* show nearly median values of the development of flexor tubercle (DFT) (*sensu* Kobayashi et al.^56^). The hypothesized outputs (HO) (*sensu* Kobayashi et al.^56^) of ungual phalanx I tend to be higher than those of other ungual phalanges (Fig. 5G). In *Hypnovenator*, ungual phalanx I is plotted near the median value of HO, while ungual phalanx III is higher than the median value of HO, resulting in both values of HO being subequal. Consequently, digit III functioned as effectively as digit I in *Hypnovenator*, whereas digit I shows greater functionality than other ungual phalanges in non-troodontine troodontids. This transition in function involves unique motions of digit III, such as immobile phalanx III-2 (also in non-named troodontid SDUST-V 1042)^46^ and the minimally flexed phalanx III-3 (also in *Deinonychus*)^46^, which may be also recognized features in other troodontines.

### Evolution of pedal structure

Three of the eleven synapomorphies for Troodontinae [characters 226, 274, 674] and one of the five synapomorphies for derived troodontines (*Urbacodon*, *Saurornithoides*, *Borogovia*, and higher taxa) [character 218] specifically pertain to the pes (Fig. 4). The asymmetrical metatarsus is characterized by a transverse width ratio of metatarsals IV to II exceeding 166% at midshaft [character 226]. Most Liaoning troodontids and *Sinornithoides* exhibit a plesiomorphic condition, with ratios of less than 120%^6,17,20,21,57^, but this increases to 158% in MPC-D 100/140 of the late Early Cretaceous troodontid^5^. More derived taxa, such as *Hypnovenator* (250%) and *Gobivenator* (272%), show a pronounced increase in metatarsal asymmetry, influencing the arctometatarsalian condition [character 219] (Supplementary Text S3). The arctometatarsalian condition is linked to the cursoriality of troodontines, supported by the weight-bearing function and the relative length of hind limbs^48,58,59^. The metatarsal arrangement in troodontines resembles those in ostriches, which possess only two pedal digits, with the midline shift between metatarsals III and IV^60^. Although troodontids have four digits, only two (digits III and IV) bear body weight due to the proximal position of digit I and the hyperextensible digit II^1^, suggesting that this similarity likely arises from convergence due to cursorial habits with only two weight-bearing digits. Non-cursorial eudromaeosaurs also have only two weight-bearing digits but the midline of metatarsus positioned on metatarsal III as in most other theropods. *Hypnovenator* is noteworthy as the oldest troodontine with an asymmetrical arctometatarsus, extending the record by 35 million years compared with previous works. In addition to the metatarsal asymmetry, there is a trend towards increased cursoriality seen in the shorter digit IV of some derived troodontines compared to non-troodontine troodontids^61^. For instance, the ratios of digits IV to III are 75% in *Troodon*, 80% in *Talos*, and 86% in *Borogovia* (assuming the non-preserved phalanx III-1 is approximately 33 mm in length based on *Saurornithoides*). These ratios are notably lower than those of the Early Cretaceous troodontids (98% in *Sinornithoides* and 96% in *Sinovenator*).

The pedal features indicative of enhanced cursoriality are expressed through the morphological characteristics of metatarsal and phalangeal articulations: ginglymoid for grasping and the absence or weakness of the intercondylar groove for cursoriality^61^. *Hypnovenator* preserves the interphalangeal articulations of digits but is missing the distal end of metatarsal III. Most non-troodontine troodontids and *Gobivenator*, a sister taxon to *Hypnovenator*, retain ginglymoid articulations to some extent for grasping, indicated by the presence of a deep distal articular groove on metatarsal III^21,23,36,47,57,62^, whereas there is no or shallow distal articular groove of metatarsal III in derived troodontines [character 218]. Derived troodontines such as *Troodon*, *Borogovia*, *Talos*, and *Saurornithoides* exhibit non-ungual interphalangeal articulation of phalanges of digit III with roller joints and phalanges of digit IV with weakly ginglymoid articulation^3,10,38,61^. *Hypnovenator*, on the other hand, displays ginglymoid articulations in the digits. The mosaic features in the pes for grasping (ginglymoid articulations in digits) and cursoriality (asymmetrical arctometatarsus) in basal troodontines suggest that *Hypnovenator,* along with *Gobivenator,* serves as a pivotal taxon, supporting a transition towards more efficient cursorial locomotion in derived troodontines.

Regarding *Hypnovenator*, although preservation issues hinder a complete assessment, members of the Troodontinae exhibit a relatively narrow trochlea of metatarsal II, defined by a transverse width ratio of metatarsals II to III trochlea less than 80% [character 674]. This narrow trochlea corresponds to the asymmetry of the metatarsus, yet its presence suggests some level of grasping ability in the hyperextensible digit II^61^. Triangular cross-sections of pedal ungual phalanges III and IV [character 274] are present in *Hypnovenator*, *Borogovia*, *Linhevenator*, and *Troodon*^10,11^. This feature is also found in non-avialan theropods except for therizinosaurs^63^, microraptorine dromaeosaurs^64^, and non-troodontine troodontids^43^, indicating a reversal in the morphology of ungual phalanges III and VI within Troodontinae, likely associated with adaptations for ground-dwelling habitats and cursoriality.

## Materials and methods

### CT-scan

To construct the holotype materials of *Hypnovenator* within host rock in three-dimension, the materials underwent microcomputed tomography (micro-CT) using a TESCO Microfocus CT TXS320-ACTIS at the National Science Museum (Tokyo, Japan), and digital images were processed and measured using Amira 2019.3 (Thermo Fisher Scientific). The complete preparation was disturbed by extremely fragile bones (gastralia and dorsal ribs) and unremovable host rock on both ends of long bones.

### Definition of taxonomic names

This study follows Sereno^65^ for the definition of Troodontidae, a stem-based monophyletic group containing *Troodon* and all coelurosaurs closer to it than *Velociraptor* or *Passer*. Sinovenatorinae was defined as the most inclusive clade including *Sinovenator* but not *Troodon*, *Saurornithoides*, *Anchiornis*, *Archaeopteryx*, *Gallus*, *Unenlagia*, or *Dromaeosaurus*^7^ and redefined as a stem-based monophyletic group containing *Sinovenator* closer to it than to *Jinfengopteryx*, *Troodon*, and *Passer*^8^. This study follows the definition of Hartman et al.^8^. Troodontinae is redefined as the least inclusive clade containing *Troodon* (when included), *Gobivenator*, and *Zanabazar* but not *Sinovenator* and *Jinfengopteryx* for the more stable clade than those of Martnuiuk^66^, van der Reest and Currie^37^, Hartman et al.^8^, Cau and Madzia^10^, and Sellés et al.^16^. This study also follows the definition of Halszkaraptorinae by Cau et al.^67^, the most inclusive clade that contains *Halszkaraptor*, but not *Dromaeosaurus*, *Unenlagia*, *Saurornithoides* or *Vultur*.

### Phylogenetic analysis

To test the phylogenetic position of *Hypnovenator* among troodontids, the data matrix of Sellés et al.^16^ was used. The broad-scale data matrix was used to resolve the phylogenetic relationship among coelurosaurian theropods including at least twenty-seven troodontid taxa. An analysis was run using the data matrix of Sellés et al.^16^, which *Hypnovenator* adds. All cordings for troodontids were checked, and some scorings were modified based on the papers and original materials (Supplementary Data S2 and Table S2). The data matrix comprises 503 taxa and 700 characters. The analysis was conducted with equally weighted parsimony using TNT v. 1.5^68^. We set the maximum number of trees saved in memory at 10,000 and used a traditional search, performing 10,000 replications of Wagner trees (using random addition sequences) followed by tree bisection reconnection (TBR) as the swapping algorithm, saving 10 trees per replicate. However, a strict consensus tree forms a polytomy within BYU 2023, ISMD-VP09, and other taxa because of the high ratio of missing data in BYU 2023 (99%). Second analysis run with the same setting as the first one excluding BYU 2023.

### Geometric morphometric analysis

Geometric morphometric analysis was performed to quantify the two-dimensional ungual phalanx morphological variations using the R package geomorph version 4.0.7^69^. Ungual outlines in lateral view were obtained from the original materials of *Hypnovenator*, MPC-D 100/140, and MPC-D 100/44, the cast of *Sinornithoides* (FPDM-V-7218), and the literatures on other troodontids^7,20,43,45,46^. *Hypnovenator* is the sole troodontine. The outlines were digitalized into four fixed landmarks and 12 sliding semi-landmarks (Supplementary Data S3–6), following Chinzorig et al.^70^. The landmarks underwent generalized Procrustes analysis^71,72^ to align the specimens. Firstly, this involved scaling all shapes (in this case, ungual phalanx landmarks) to a uniform size, followed by rotating the shape coordinates around the origin to minimize shape differences. Subsequently, principal component analysis (PCA) was applied to the covariance matrix of the Procrustes coordinates. PCA identifies maximum variance in multidimensional datasets, summarizing the original data as PC1, PC2, and so on. Consequently, PCA facilitates the graphical representation of multivariate data in a two-dimensional graph, as shown in Fig. 5^73,74^. To evaluate the functionality of troodontid ungual phalanges, mechanical advantage (MA) was computed. As MA corresponds to a class 3 lever^75^, resultant MA values indicate the proportion of output force exerted on the ungual phalanx tip relative to the input force at the flexor tubercle.

$${\text{MA }} = {\text{ sin }}\left( {\theta \, + \, \delta } \right){\text{ d}}/{\text{a}},$$

The mechanical advantage of the ungual phalanx can be determined using above equation, where 'a' represents the output lever length from the fulcrum to the resistance, 'd' denotes the length from the fulcrum to the flexor tubercle, 'θ' signifies the angle of the input force vector to the line of output lever, and 'δ' represents the angle between the line from the fulcrum to the flexor tubercle and the line of output lever^69^. The size of the flexor tubercle is closely associated with the cross-sectional area of the attached muscle, correlating with the maximum input force. Thus, the flexor tubercle size was quantified as a ratio of the perpendicular length from the apex of the flexor tubercle to the segment between the base of the flexor tubercle, serving as a proxy of the input force. Multiplying the flexor tubercle size by the mechanical advantage yields the inferred output force at the tip. These inferred output forces were compared across digits I to III. Standardized major axis (SMA) regression analyses, utilizing R package smatr version 3.4.8, were employed to examine the relationship between the obtained PC scores and the inferred output force, thereby assessing the shape-function relationships of troodontid ungual phalanges. All statistical analyses were conducted on software R version 4.3.2^76^ using the R script, with modifying file names, provided by Kobayashi et al.^56^.

### Supplementary Information

Supplementary Information 1.

Supplementary Information 2.

Supplementary Information 3.

Supplementary Information 4.

Supplementary Information 5.

## Data Availability

All data generated or analyzed during this study are included in this published article and its Supplementary Information files.
