# Supplementary material for: Early Cretaceous troodontine troodontid (Dinosauria: Theropoda) from the Ohyamashimo Formation of Japan reveals the early evolution of Troodontinae
Source: Sci Rep. 2024 Jul 25;14:16392. doi: 10.1038/s41598-024-66815-2 (PMC11272788; doi:10.1038/s41598-024-66815-2)
Supplement: Supplementary file 5 — Supplementary Information 5. [file 41598_2024_66815_MOESM5_ESM.docx]

Supplementary information for “EARLY CRETACEOUS TROODONTINE TROODONTID (DINOSAURIA: THEROPODA) FROM THE OHYAMASHIMO FORMATION OF JAPAN REVEALS THE EARLY EVOLUTION OF TROODONTINAE” by Katsuhiro Kubota, Yoshitsugu Kobayashi, and Tadahiro Ikeda

**Supplementary Text**

Supplementary Text S1. Troodontids in Japan.

Some trace fossils with the potential to show the presence of troodontids in Japan have been recently recovered. Two prismatoolithid eggshells, *Ramoprismatoolithus okurai* and *Prismatoolithus* oosp., have been reported from the lower Hauterivian Okurodani Formation of the Tetori Group and the lower Albian Ohyamashimo Formation of the Sasayama Group, respectively^1-3^. Although *Prismatoolithus* has been associated with troodontid remains^4,5^, recent phylogenetic analyses of oological traits suggest that *Prismatoolithus* belongs not only to troodontids but also to avian taxa^6,7^. Two of the three cladograms in Uematsu *et al*.^3^ show that *Ramoprismatoolithus okurai* is attributable to troodontids or closely related non-avian maniraptorans, another cladogram forms a polytomy with non-avian maniraptorans and avian groups. A deinonychosaur footprint, Dromaeopodidae ichnogen. et ichnosp. indet., is found in the Aptian Kitadani Formation of the Tetori Group, but it cannot conclusively indicate the presence of troodontids because Deinonychosauria contains both Troodontidae and Dromaeosauridae^8^. *Paronychodon* teeth, a *nomen dubium* taxon once referred to as Troodontidae, were recovered from the Hauterivian to Barremian and Turonian deposits in Japan^9,10^. These teeth bear longitudinal ridges on the crown surface and resemble the premaxillary teeth of dromaeosaurids, such as *Bambiraptor feinbergi*, *Saurornitholestes langstoni*, and *Velociraptor mongoliensis*, rather than troodontids^11^. Consequently, the discovery of *Hypnovenator matsubaraetoheorum* represents the first unambiguous evidence of a troodontid in Japan and the participation of a troodontid in the dinosaur assemblage of Japan by the early to middle Albian.

References for Supplementary Text S1

1 Tanaka, K. *et al*. Dinosaur eggshell assemblage from Japan reveals unknown diversity of small theropods. *Cretac. Res.* **57**, 350-363; [10.1016/j.cretres.2015.06.002](https://doi.org/10.1016/j.cretres.2015.06.002) (2016).

2 Tanaka, K. *et al*. Exceptionally small theropod eggs from the Lower Cretaceous Ohyamashimo Formation of Tamba, Hyogo Prefecture, Japan. *Cretac. Res.* **114**, e104519; [10.1016/j.cretres.2020.104519](https://doi.org/10.1016/j.cretres.2020.104519) (2020).

3 Uematsu, R. *et al*. Fossil eggshells from the Early Cretaceous Okurodani Formation, northern central Japan. *Hist. Biol.*; [10.1080/08912963.2022.2142910](https://doi.org/10.1080/08912963.2022.2142910) (2022).

4 Varricchio, D. J., Jackson, F., Borkowski, J. J. & Horner, J. R. Nest and egg clutches of the dinosaur *Troodon formosus* and the evolution of avian reproductive traits. *Nature* **385**(6613), 247–250; [10.1038/385247a0](https://doi.org/10.1038/385247a0) (1997).

5 Varricchio, D. J., Horner, J. R. & Jackson, F. D. Embryos and eggs for the Cretaceous theropod dinosaur *Troodon formosus*. *J. Vertebr. Paleontol.* **22**(3), 564–576; [10.1671/0272-4634(2002)022[0564:EAEFTC]2.0.CO;2](https://doi.org/10.1671/0272-4634(2002)022%5b0564:EAEFTC%5d2.0.CO;2) (2002).

6 Moreno-Azanza, M., Canudo, J. I. & Gasca, J. M. Unusual theropod eggshells from the Early Cretaceous Blesa Formation of the Iberian Range, Spain. *Acta Palaeontol. Pol.* **59**(4), 843–854; [10.4202/app.2012.0069](https://doi.org/10.4202/app.2012.0069) (2014).

7 Vila, B., Sellés, A. G. & Beetschen, J. C. The controversial Les Labadous eggshells: a new and peculiar dromaeosaurid (Dinosauria: Theropoda) ootype from the Upper Cretaceous of Europe. *Cretac Res.* **72**,117-123; [10.1016/j.cretres.2016.12.010](https://doi.org/10.1016/j.cretres.2016.12.010) (2017).

8 Tsukiji, Y., Hattori, S. & Azuma, Y. New theropod track from the Kitadani Formation of Katsuyama City, Fukui Prefecture. *Abstr. 172th Regul. Meeting Palaeontol. Soc. Japan*, 26 (2023).

9 Kurosu, M., Hirayama, R., Yoshida, M. & Takekawa, A. Classification and comparison of theropods from the Upper Cretaceous Kuji Group, Iwate Prefecture. *Abstr. 166th Regul. Meeting Palaeontol. Soc. Japan*, 16 (2017).

10 Kubota, K. A list of Mesozoic dinosaur fossils from Japan in 2022. *Bull. Gunma Mus. Nat. Hist.* **27**, 53-66 (2023).

11 Currie, P. J. & Evans, D. C. Cranial anatomy of new specimens of *Saurornitholestes langstoni* (Dinosauria, Theropoda, Dromaeosauridae) from the Dinosaur Park Formation (Campanian) of Alberta. *The Anatomical Record* **303**(4), 691–715; [10.1002/ar.24241](https://doi.org/10.1002/ar.24241) (2020).

Supplementary Text S2. The line of evidences that theropod bones found at Nishikosa in 2010 and 2011 are assigned to a single individual skeleton.

Two blacks, each containing theropod bones, were discovered at the same locality in Nishikosa, Tambasasayama City, Hyogo Prefecture in 2010 (Fig. 2A) and 2011 (Fig. 2B), respectively, where part of the crushed strata for constructing the public park was accumulated. Both blocks are commonly composed of poorly sorted and grayish-brown muddy sandstone with plant fragments, suggesting that these likely originated from the same horizon. There is no overlapping in bone content between the two blocks. Only the tibia is preserved in isolation, with the proximal and distal parts included in each block, but the mid-shaft is missing. In *Hypnovenator matsubaraetoheorum*, the ratio of the anteroposterior heights of the tibia at the fibular crest to the distal tip of the ascending process of the astragalus is 98%. Because the value is similar to those of other troodontids (e.g., 94% in *Liaoningvenator curriei*^1^), it is most likely that both parts of the tibia belong to a single bone. These potential pieces of evidence suggest that the theropod bones separately included in each block are parts of a single individual skeleton.

References for Supplementary Text S2

1 Shen, C. *et al*. A new troodontid dinosaur (*Liaoningvenator curriei* gen. et sp. nov.) from the Early Cretaceous Yixian Formation in western Liaoning province. *Acta Geosci. Sin.* **38**(3), 359-371; [10.3975/cagsb.2017.03.06](https://doi.org/10.3975/cagsb.2017.03.06) (2017).

Supplementary Text S3. Timings of asymmetrical metatarsus and arctometatarsalian condition.

Our phylogenetic analysis shows the arctometatarsalian condition as a synapomorphy for a monophyly for a subclade of *Daliansaurus* and four Late Cretaceous taxa, and a subclade of *Geminiraptor* and higher taxa, as well as convergence in MPC-D 100/1128 [character 219] (Fig. 4A, F). Similarly, the asymmetrical metatarsus is considered a synapomorphy for two clades, the subclade of *Daliansaurus* and four Late Cretaceous taxa, and Troodontinae, but also as convergence in MPC-D 100/1128 [character 226] (Fig. 4C, F). The inconsistent distribution of these characters is partly due to undecided scorings (1/2) of character 219 in *Sinornithoides*^1^ and MPC-D 100/140^2^, which show less than 165% in a ratio of metatarsals IV to II. Although *Daliansaurus* appears to have an asymmetrical metatarsus^3^, post-burial deformation has affected the high ratio of metatarsals II to IV. Consequently, the asymmetrical arctometatarsus occurs with certainty only in *Hypnovenator* and the Late Cretaceous troodontids.

References for Supplementary Text S3

1 Currie, P. J. & Dong, Z. New information on Cretaceous troodontids (Dinosauria, Theropoda) from the People's Republic of China. *Can. J. Earth Sci.* **38**(12), 1753-1766; [10.1139/e01-06](https://doi.org/10.1139/e01-065) (2001).

2 Tsuihiji, T. *et al*. New material of a troodontid theropod (Dinosauria: Saurischia) from the Lower Cretaceous of Mongolia. *Hist. Biol.* **28**(1-2), 128-138; [10.1080/08912963.2015.1005086](https://doi.org/10.1080/08912963.2015.1005086) (2016).

3 Shen, C. *et al*. A New Troodontid Dinosaur from the Lower Cretaceous Yixian Formation of Liaoning Province, China. *Acta Geol. Sin. (English Edition)* **91**(3), 763-780; [10.1111/1755-6724.13307](https://doi.org/10.1111/1755-6724.13307) (2017).

**Supplementary Table**

Supplementary Table S1. Measurement of *Hypnovenator matsubaraetoheorum* gen. et sp. nov. The length of manual phalanx II-2 is estimated based on the distance between the distal end of phalanx II-1 and the proximal articular surface of phalanx II-3. * Estimated value; + preserved minimum value.

| Unit: mm | Length | proximal end | | mid-shaft | | distal end | |
| --- | --- | --- | --- | --- | --- | --- | --- |
|  |  | height | width | height | width | height | width |
| Humerus | 77.4+ | 25.1+ | ? | 5.9 | 5.7 | 5.0 | 13.1 |
| Radius | 73.4 | 3.5 | 4.7 | 2.3 | 2.8 | 3.4 | 4.8 |
| Ulna | 76.1 | 6.1 | 7.7 | 3.3 | 2.9 | 5.7 | 8.4 |
| Semilunate carpal | 3.3 | -- | -- | -- | -- | 6.7 | 7.5 |
| Metacarpal I | 14.3 | 3.4 | 4.2 | 3.9 | 3.2 | 4.2 | 4.4 |
| Metacarpal II | 35.8 | 3.6 | 4.3 | 3.5 | 2.4 | 5.1 | 4.5 |
| Metacarpal III | 34.4 | 2.3 | 2.7 | 1.9 | 1.6 | 3.1 | 2.5 |
| Manual phalanx I-1 | 31.6 | 5.9 | 4.2 | 2.4 | 2.5 | 4.2 | 3.0 |
| Manual phalanx I-2  (manual ungual phalanx I) | 19.7 | 5.5 | 3.0 | -- | -- | -- | -- |
| Manual phalanx II-1 | 22.0 | 6.0 | 5.2 | 2.4 | 2.1 | 4.0 | 3.5 |
| Manual phalanx II-2 | 28* | ? | ? | ? | ? | ? | ? |
| Manual phalanx II-3  (manual ungual phalanx II) | 17.2+ | ? | ? | -- | -- | -- | -- |
| Manual phalanx III-1 | 8.3 | 4.5 | 3.1 | 2.9 | 2.1 | 2.3 | 2.4 |
| Manual phalanx III-2 | 9.0 | 3.9 | 2.5 | 2.8 | 2.1 | 2.6 | 2.7 |
| Manual phalanx III-3 | 22.8 | 4.2 | 3.1 | 2.2 | 1.6 | 2.9 | 2.3 |
| Manual phalanx III-4  (manual ungual phalanx III) | 13.2 | 4.2 | 2.1 | -- | -- | -- | -- |
| Femur | 46.1+ | ? | ? | ? | ? | 18.5 | 20.1 |
| Tibia proximal | 32.8+ | 25.8 | 14.1 | 7.4 | 9.0 | -- | -- |
| Tibia distal | 58.8+ | -- | -- | 7.7 | 8.7 | 7.6 | 16.9 |
| Fibula proximal | 30.2+ | 17.9 | 5.1 | ? | ? | -- | -- |
| Fibula distal | 60.6+ | -- | -- | 0.8 | 1.0 | 1.7 | ? |
| Astragalus | 29.4 | 12.2 | 16.9 | -- | -- | -- | -- |
| Metatarsal II | 60.3+ | 10.5 | 7.0 | 5.3 | 2.2 | ? | ? |
| Metatarsal III | 57.2+ | 5.7 | 3.6 | 1.2 | ? | ? | ? |
| Metatarsal IV | 61.1+ | 10.1 | 9.3 | 8.1 | 5.5 | ? | ? |
| Pedal phalanx II-3  (pedal ungual phalanx II) | 15.5+ | ? | ? | -- | -- | -- | -- |
| Pedal phalanx III-1 | 15.1+ | ? | ? | 3.3 | 3.7 | 5.4 | 6.1 |
| Pedal phalanx III-2 | 15.3 | 6.9 | 5.6 | 2.9 | 3.2 | 4.7 | 4.7 |
| Pedal phalanx III-3 | 15.4 | 5.2 | 4.8 | 2.0 | 3.1 | 4.2 | 3.9 |
| Pedal phalanx III-4  (pedal ungual phalanx III) | 11.9 | 4.1 | 2.9 | -- | -- | -- | -- |
| Pedal phalanx IV-1 | 4.9+ |  |  |  |  | 4.8 | 5.5 |
| Pedal phalanx IV-2 | 12.7 | 6.9 | 5.4 | 2.8 | 3.5 | 4.1 | 4.9 |
| Pedal phalanx IV-3 | 11.2 | 5.5 | 5.0 | 2.3 | 3.3 | 3.5 | 4.5 |
| Pedal phalanx IV-4 | 11.4 | 4.7 | 3.8 | 1.6 | 3.0 | 4.1 | 3.9 |
| Pedal phalanx IV-5  (pedal ungual phalanx IV) | 11.7 | 4.5 | 3.4 | -- | -- | -- | -- |

Supplementary Table S2. List of troodontid taxa used in the comparative study and phylogenetic analysis.

| **Taxon** | | **Source** |
| --- | --- | --- |
|  | *Almas ukhaa* | Pei *et al*.^1^ |
|  | *Borogovia gracilicrus* | Osmólska^2^; Cau and Madzia^3^ |
|  | *Byronosaurus jaffei* | MPC-D 100/983; MPC-D 100/984; Norell *et al*.^4^; Makovicky *et al*.^5^ |
|  | *Daliansaurus* *liaoningensis* | Shen *et al*.^6^ |
|  | *Geminiraptor suarezarum* | Senter *et al*.^7^ |
|  | *Gobivenator mongoliensis* | MPC-D 100/86; Tsuihiji *et al*.^8^ |
|  | *Hesperornithoides miessleri* | Hartman *et al*.^9^ |
|  | *Jianianhualong tengi* | Xu *et al*.^10^ |
|  | *Jinfengopteryx* *elegans* | Ji *et al*.^11^ |
|  | *Liaoningvenator curriei* | Shen *et al*.^12^ |
|  | *Linhevenator tani* | Xu *et al*.^13^ |
|  | *Mei long* | IVPP V12733; Xu *et al*.^14,16^; Gao *et al*.^15^ |
|  | *Papiliovenator neimengguensis* | Pei *et al*.^17^ |
|  | *Philovenator curriei* | Currie and Peng^18^; Xu *et al*.^19^ |
|  | *Saurornithoides mongoliensis* | AMNH 6516; Osborn^20^; Norell *et al*.^21^ |
|  | *Sinornithoides youngi* | FPDM-V-7218 (a cast of IVPP V9612); Russell and Dong^22^; Currie and Dong^23^ |
|  | *Sinovenator changii* | Xu *et al*.^16,24^; White^25^; Yin *et al*.^26^ |
|  | *Sinusonasus magnodens* | Xu and Wang^27^ |
|  | *Talos sampsoni* | Zanno *et al*.^28^ |
|  | *Tamarro insperatus* | Sellés *et al*.^29^ |
|  | *Tochisaurus nemegtensis* | Kurzanov and Osmólska^30^ |
|  | *Troodon formosus*  (= *Latenivenatrix mcmasterae*) | NMC 1650; NMC 12340; TMP 1979.008.0001; TMP 1986.036.0004; TMP 1992.36.575; Sternberg^31^; Russell^32^; van der Reest and Currie^33^ |
|  | *Urbacodon itemirensis* | Averianov and Sues^34^ |
|  | *Urbacodon* sp. | Averianov and Sues^35^ |
|  | *Xiaotingia zhengi* | Xu *et al*.^36^ |
|  | *Xixiasaurus henanensis* | Lü *et al*.^37^ |
|  | *Zanabazar junior* | MPC-D 100/1; Norell *et al*.^21^; Barsbold^38^ |
|  | IVPP V11119 | Dong^39^ |
|  | MPC-D 100/44 | MPC-D 100/44; Barsbold *et al*.^40^ |
|  | MPC-D 100/140 | MPC-D 100/140; Tsuihiji *et al*.^41^ |
|  | MPC-D 100/983 and 100/984 | Bever and Norell^42^ |
|  | SDUST V142 | Yu *et al*.^43^ |

References for Supplementary Table S2

1. Pei, R. *et al*. Osteology of a new Late Cretaceous troodontid specimen from Ukhaa Tolgod, Ömnögovi Aimag, Mongolia. *Am. Mus. Novit.* **3889**, 1-47; [10.1206/3889.1](https://doi.org/10.1206/3889.1) (2017).
2. Osmólska, H. *Borogovia gracilicrus* gen. et sp. n., a new troodontid dinosaur from the Late Cretaceous of Mongolia. *Act. Palaeontol. Pol.* **32**(1-2), 133-150 (1987).
3. Cau, A. & Madzia, D. The phylogenetic affinities and morphological peculiarities of the bird-like dinosaur *Borogovia gracilicrus* from the Upper Cretaceous of Mongolia. *PeerJ* **9**, e12640; [10.7717/peerj.12640](http://doi.org/10.7717/peerj.12640) (2021).
4. Norell, M. A., Makovicky, P. J. & Clark, J. M. A new troodontid theropod from Ukhaa Tolgod, Mongolia. *J. Vertebr. Paleontol.* **20**(1), 7-11; [10.1671/0272-4634(2000)020[0007:ANTTFU]2.0.CO;2](https://doi.org/10.1671/0272-4634(2000)020%5b0007:ANTTFU%5d2.0.CO;2) (2000).
5. Makovicky, P. J., Norell, M. A., Clark, J. M. & Rowe, T. Osteology and relationships of *Byronosaurus jaffei* (Theropoda: Troodontidae). *Am. Mus. Novit.* **3402**, 1-32; [10.1206/0003-0082(2003)402<0001:OAROBJ>2.0.CO;2](https://doi.org/10.1206/0003-0082(2003)402%3c0001:OAROBJ%3e2.0.CO;2) (2003).
6. Shen, C. *et al*. A New Troodontid Dinosaur from the Lower Cretaceous Yixian Formation of Liaoning Province, China. *Acta Geol. Sin. (English Edition)* **91**(3), 763-780; [10.1111/1755-6724.13307](https://doi.org/10.1111/1755-6724.13307) (2017).
7. Senter, P., Kirkland, J. I., Bird, J. & Bartlett, J. A. A New Troodontid Theropod Dinosaur from the Lower Cretaceous of Utah. *PloS ONE* **5**(12), e14329; [10.1371/journal.pone.0014329](https://doi.org/10.1371/journal.pone.0014329) (2010).
8. Tsuihiji, T. *et al*. An exquisitely preserved troodontid theropod with new information on the palatal structure from the Upper Cretaceous of Mongolia. *Naturwiss.* **101**, 131-142; [10.1007/s00114-014-1143-9](https://doi.org/10.1007/s00114-014-1143-9) (2014).
9. Hartman, S. *et al*. A new paravian dinosaur from the Late Jurassic of North America supports a late acquisition of avian flight. *PeerJ* **7**, e7247; [10.7717/peerj.7247](https://doi.org/10.7717/peerj.7247) (2019).
10. Xu, X. *et al*. Mosaic evolution in an asymmetrically feathered troodontid dinosaur with transitional features. *Nat. Commun.* **8**(1), 1-12; [10.1038/ncomms14972](https://doi.org/10.1038/ncomms14972) (2017).
11. Ji, Q. *et al*. First avialian bird from China (*Jinfengopteryx elegans* gen. et sp. nov.). *Geol. Bull. Chi.* **24**(3), 197-210 (2005).
12. Shen, C. *et al*. A new troodontid dinosaur (*Liaoningvenator curriei* gen. et sp. nov.) from the Early Cretaceous Yixian Formation in western Liaoning province. *Acta Geosci. Sin.* **38**(3), 359-371; [10.3975/cagsb.2017.03.06](https://doi.org/10.3975/cagsb.2017.03.06) (2017).
13. Xu, X. *et al*. A short-armed troodontid dinosaur from the Upper Cretaceous of Inner Mongolia and its implications for troodontid evolution. *PLoS ONE* **6**(9), e22916; [10.1371/journal.pone.0022916](https://doi.org/10.1371/journal.pone.0022916) (2011).
14. Xu, X. & Norell, M. A. A new troodontid dinosaur from China with avian-like sleeping posture. *Nature* **431**(7010), 838-841; [10.1038/nature02898](https://doi.org/10.1038/nature02898) (2004).
15. Gao, C., Morschhauser, E. M., Varricchio, D. J., Liu, J. & Zhao, B. A second soundly sleeping dragon: new anatomical details of the Chinese troodontid Mei long with implications for phylogeny and taphonomy. *PLoS ONE* **7**(9), e45203; [10.1371/journal.pone.0045203](https://doi.org/10.1371/journal.pone.0045203) (2012).
16. Xu, X., Han, F. & Zhao, Q. Homologies and homeotic transformation of the theropod ‘semilunate’ carpal. *Sci. Rep.* **4**, e6042; [10.1038/srep06042](https://doi.org/10.1038/srep06042) (2014).
17. Pei, R. *et al*. A New Troodontid from the Upper Cretaceous Gobi Basin of Inner Mongolia, China. *Cretac. Res.* **130**, 105052; [10.1016/j.cretres.2021.105052](https://doi.org/10.1016%2Fj.cretres.2021.105052) (2022).
18. Currie, P. J. & Peng, J. -H. A juvenile specimen of *Saurornithoides mongoliensis* from the Upper Cretaceous of northern China. *Can. J. Earth Sci.* **30**, 2224-2230; [10.1139/e93-193](https://doi.org/10.1139/e93-193) (1993).
19. Xu, X. *et al*. The taxonomy of the troodontid IVPP V 10597 reconsidered. *Vert. PalAs.* **50**, 140-150 (2012).
20. Osborn, H. F. Three new Theropoda, *Protoceratops* Zone, Central Mongolia. *Am. Mus. Novit.* **144**, 1-12 (1924).
21. Norell, M. A. *et al*. A Review of the Mongolian Cretaceous Dinosaur *Saurornithoides* (Troodontidae: Theropoda). *Am. Mus. Novit.* **3654**, 1-63; [10.1206/648.1](https://doi.org/10.1206/648.1) (2009).
22. Russell, D. A. & Dong Z. -M. A nearly complete skeleton of a new troodontid dinosaur from the Early Cretaceous of the Ordos Basin, Inner Mongolia, People’s Republic of China. *Can. J. Earth Sci.* **30**, 2163-2173; [10.1139/e93-187](https://doi.org/10.1139/e93-187) (1993).
23. Currie, P. J. & Dong, Z. New information on Cretaceous troodontids (Dinosauria, Theropoda) from the People's Republic of China. *Can. J. Earth Sci.* **38**(12), 1753-1766; [10.1139/e01-06](https://doi.org/10.1139/e01-065)5 (2001).
24. Xu, X. *et al*. A basal troodontid from the Early Cretaceous of China. *Nature* **415**, 780-784; [10.1038/415780a](https://doi.org/10.1038/415780a) (2002).
25. White, M. A. The subarctometatarsus: intermediate metatarsus architecture demonstrating the evolution of the arctometatarsus and advanced agility in theropod dinosaurs. *Alcheringa* **33**, 1-21; [10.1080/03115510802618193](https://doi.org/10.1080/03115510802618193) (2009).
26. Yin, Y. -L., Pei, R. & Zhou, C. -F. Cranial morphology of *Sinovenator changii* (Theropoda: Troodontidae) on the new material from the Yixian Formation of western Liaoning, China. *Peer J* **6**, e4977; [10.7717/peerj.4977](https://doi.org/10.7717/peerj.4977) (2018).
27. Xu, X. & Wang, X. A new Troodontid (Theropoda: Troodontidae) from the Lower Cretaceous Yixian Formation of Western Liaoning, China. *Acta Geol. Sin.* **78**(1), 22-26; [10.1111/j.1755-6724.2004.tb00671.x](https://doi.org/10.1111/j.1755-6724.2004.tb00671.x) (2004).
28. Zanno, L. E. *et al*. A New Troodontid Theropod, *Talos sampsoni* gen. et sp. nov., from the Upper Cretaceous Western Interior Basin of North America. *PLoS ONE* **6**, e24487; [10.1371/journal.pone.0024487](https://doi.org/10.1371/journal.pone.0024487) (2011).
29. Sellés, A. G. *et al*. A fast-growing basal troodontid (Dinosauria: Theropoda) from the latest Cretaceous of Europe. *Sci. Rep.* **11**(1), 4855; [10.1038/s41598-021-83745-5](https://doi.org/10.1038/s41598-021-83745-5) (2021).
30. Kurzanov, S. M. & Osmólska, H. *Tochisaurus nemegtensis* gen. et sp. n., a new troodontid (Dinosauria, Theropoda) from Mongolia. *Acta Palaeontol. Pol.* **36**, 69-76 (1991).
31. Sternberg, C. M. Two new theropod dinosaurs from the Belly River Formation of Alberta. *The Canadian Field-Naturalist* **46**(5), 99-105 (1932).
32. Russell, D. A. A new specimen of *Stenonychosaurus* from the Oldman Formation (Cretaceous) of Alberta. *Can. J. Earth Sci.* **6**, 595-612; [10.1139/e69-059](https://doi.org/10.1139/e69-059) (1969).
33. van der Reest, A. J. & Currie, P. J. Troodontids (Theropoda) from the Dinosaur Park Formation, Alberta, with a description of a unique new taxon: implications for deinonychosaur diversity in North America. *Can. J. Earth Sci.* **54**(9), 919-935; [10.1139/cjes-2017-00](https://doi.org/10.1139/cjes-2017-0031)31 (2017).
34. Averianov, A.O. & Sues, H.-D. A new troodontid (Dinosauria: Theropoda) from the Cenomanian of Uzbekistan, with a review of troodontid records from the territories of the former Soviet Union. *J. Vertebr. Paleontol.* **27**, 87-98; [10.1671/0272-4634(2007)27[87:ANTDTF]2.0.CO;2](https://doi.org/10.1671/0272-4634(2007)27%5b87:ANTDTF%5d2.0.CO;2) (2007).
35. Averianov, A. O. & Sues, H. -D. Troodontidae (Dinosauria: Theropoda) from the Upper Cretaceous of Uzbekistan. *Cretac. Res.* **59**, 98-110; [10.1016/j.cretres.2015.11.005](https://doi.org/10.1016/j.cretres.2015.11.005) (2016).
36. Xu, X., You, H., Du, K. & Han, F. An *Archaeopteryx*-like theropod from China and the origin of Avialae. *Nature* **475**, 465-470; [10.1038/nature10288](https://doi.org/10.1038/nature10288) (2011).
37. Lü, J. -C. *et al*. A new troodontid theropod from the Late Cretaceous of central China, and the radiation of Asian troodontids. *Acta Palaeontol. Pol.* **55**, 381-388; [10.4202/app.2009.0047](https://doi.org/10.4202/app.2009.0047) (2010).
38. Barsbold, R. Saurornithoidedae, a new family of small theropod dinosaurs from central Asia and North America. *Palaeontol. Pol.* **30**, 5-22 (1974).
39. Dong, Z. On Small Theropoda from Mazongshan Area, Gansu Province, China. In *Sino-Japanese Silk Road Dinosaur Expedition* (ed Dong, Z.) 13-18 (China Ocean Press, 1997).
40. Barsbold, R., Osmóska, H. & Kurzanov, S. M. On a new troodontid (Dinosauria, Theropoda) from the Early Cretaceous of Mongolia. *Acta Palaeontol. Pol.* **32**(1-2), 121-132 (1987).
41. Tsuihiji, T. *et al*. New material of a troodontid theropod (Dinosauria: Saurischia) from the Lower Cretaceous of Mongolia. *Hist. Biol.* **28**(1-2), 128-138; [10.1080/08912963.2015.1005086](https://doi.org/10.1080/08912963.2015.1005086) (2016).
42. Bever, G. & Norell, M. A. The Perinate Skull of Byronosaurus (Troodontidae) with Observations on the Cranial Ontogeny of Paravian Theropods. *Am. Mus. Novit.* **3657**, 1-51; [10.1206/650.1](https://doi.org/10.1206/650.1) (2009).
43. Yu, D., Pei, R., Yin, Y.-L. & Zhou, C.-F. The morphology and function of the manual digits of a troodontid from the Yixian Formation of western Liaoning, China. *Hist. Biol.* **36**(1), 183-192; [10.1080/08912963.2022.2155149](https://doi.org/10.1080/08912963.2022.2155149) (2024).

**Supplementary Data**

Supplementary Data S1. List of synapomorphies supporting nodes shared by all MPTs in the analysis. Character numbers and descriptions refer to Sellés *et al*.^1^. Node numbers are shown in Supplementary Fig. S15. Numbers highlighted by green colors show the characters on pes.

**Node 1**: 50 (1->0), 169 (1->0), 212 (0->1), 237 (1->0), 267 (2/3->1), 388 (0->1), 657 (0->1)

**Node 2**: 117 (0->1), 248 (2->1), 358 (1->0), 520 (1->0)

**Node 3**: 672 (0->1), 675 (1->0)

**Node 4**: 27 (0->1), 42 (0->1), 65 (1->0), 104 (0->1), 126 (1->2), 247 (1->0), 268 (0->1), 306 (1->2), 380 (1->2), 392 (2->3)

**Node 5**: 6 (1->0), 47 (1->0), 91 (1->2), 213 (0->1), 302 (0->1), 486 (0->1), 518 (1->0), 573 (0->1)

**Node 6**: 17 (1->0), 167 (1->0)

**Node 7**: 80 (1->2), 224 (0->1)

**Node 8**: 5 (0->1), 83 (0->1), 180 (0->1), 241 (0->1), 261 (0->1), 263 (0->1), 265 (0->1), 640 (0->1)

**Node 9**: 50 (0->1), 310 (0->1), 467 (0->1), 491 (1->2)

**Node 10**: 4 (3->1/2), 44 (2->1), 101 (0->1), 131 (0->1), 149 (0->1), 167 (0->1), 168 (0->1), 306 (2->1)

**Node 11**: 182 (1->0), 197 (1->2), 248 (2->1), 281 (0->1), 302 (0->1), 378 (0->1)

**Node 12**: 77 (1->0), 227 (1->0), 229 (2->1), 310 (0->1)

**Node 13**: 92 (1->0), 219 (1->2), 377 (0->1)

**Node 14**: 204 (0->1), 226 (1->2), 657 (1->0)

**Node 15**: 7 (1->0), 77 (1->0), 301 (0->1)

**Node 16**: 340 (0->1)

**Node 17**: 326 (1->0)

**Node 18**: 268 (1->0)

**Node 19**: 114 (0->1), 267 (1->0), 378 (1->0)

**Node 20**: 7 (1->0), 176 (1->0), 183 (2->1), 191 (1->0), 192 (2->1), 197 (2->1), 226 (1->2), 227 (1->0), 274 (1->0), 473 (0->1), 674 (1->0)

**Node 21**: 152 (1->0), 209 (1->0), 212 (1->0), 256 (0->1)

**Node 22**: 6 (1->0), 30 (0->1), 218 (1->0), 241 (0->1), 244 (2->1)

**Node 23**: 666 (0->1)

**Node 24**: 206 (1->0)

**Node 25**: 42 (1->0), 63 (1->0), 314 (1->0)

References for Supplementary Data S1

1 Sellés, A. G. *et al*. A fast-growing basal troodontid (Dinosauria: Theropoda) from the latest Cretaceous of Europe. *Sci. Rep.* **11**(1), 4855; [10.1038/s41598-021-83745-5](https://doi.org/10.1038/s41598-021-83745-5) (2021).

Supplementary Data S2. Phylogenetic dataset of Sellés *et al*.^1^ for troodontids used in the analyses of this study. Underbarred numbers show the scorings modified based on the papers and original materials shown in Supplementary Table S2.

*Mei* ?[01]0[12]0??010?????????0???????0100??101???00101021101101?010001??110?????0?0[01]0[01]100?1?1100002[123]1??0?2??0011011110201000100???01100201111011???11[01]101102001011?1000000000010110122111?10[01]111200031111?1000111100000100101?[01]100001[01]0200011020?[01]000110??[01]?01[01]1[01]2?100?100210010001?0?100?01????100?0000?110100?0?0000[01]?10111??1[01]0[012]1111221?0?00101?0111101201?002[01]01101??0?10201?021?10111?100020000101?1?0?11000300100001[01]110?0?00000?0?001101000210?0??01?001?0?1?0?00????00110100101?[01]0??10101[01][01]0??0?0??100000?00200?0000?0001?0?1000??0?211?1??1?00?00?01000?0000200000???????00????0??00?0???????000?[01]0100?00?0???????000?000000?0?0001?1?0000?00?00100101210?00000?1?00000001001000??00002?110000000011??0????1101000111??0000?0

*Xiaotingia* ?0?01110????????????????????1????1?0????0?010???0???????001???????????0?000?0??2??000??020[12]??022???0110???1?[02]????2[01]00??00?10??????10?0????1110??120010???100000000001111?2??1102?0[01]?222000?[12]?[12]??1[12]?????1??0?1??????????[01]??[123]00[23]0101????000????1??1?0[12]1?0??1?1?2?11210??1?101010?12?1????00?0000?110?10?0?0?101??0?100???0?1111?[12]?00????0????0?1??201000200?10??????0?[12]1?021?0?111000002[01]000121?10[01]?1?000[23]000000???0?0??100?0????0?11??0??????0??0????1?????0?1???????1001?110??0???101[01][01]???000?0??1??000??0????10???0?0???1???0??0???????01?01?0?10?1???001?1??000????????????????00?????????00??0????0?0?0???????001???????????1010??1?00??0?00?00???2?0?0000??1[01]0?000???00?0??0?1??????????????[01]11?0????0????00?10?????00??

*Sinovenator* ?10311101?0001010?[01]0000111110001?0?0?0000102021101?1100100011000111001100101101[12]10100000[12]211?002011?0101?1110?001[01]?0010001100201110???????1110111?0000???10000?00000[01]00001?211011001?220003111121[12]0?111100000100101?[01]0000110???1011020100?[01]000111?021102??0101?01???10101010100?1????000?100001110000[01]00?0000001021001000111122100000200001001?1201000200010110010021[01]??21?10101????????000?2?110111??030010?001?0100010000?0??0011010002???1?001?001000000?000000001?0??110??111?1010120?0100001?000?0?0020??100?1000100?10110??0101?11?1100?00101?0???000300000000?00000??0000?00100?0????0000[012]0100?01?0???????????000000?0100010??100???0?00?1????????0???0????00000010?00000100000000?0?000?011?000100100???????0000?001

*Jianianhualong* ??0[123]1?00100?????????????????????000?1??001?20[12]??00011???0????????1?0??[01]0010010021?1????01[12]11?002?????1????0??????0?0???0?11??[12]011[01]000??????1?01??20000??1100100000001000???????????1??200?3[12]1[12]??1?0???0????0??????????00001?020??1???????0?1?1??1?0[23]1??2??0??1?011001?101?1?0?10??110?[01]00??0?0???01?0?0?????0?00?[12]10?00?011111??00000[01]010?1??1?12?10002001?0??00??0211??11?1011?1?00?2001012??1?0?101002?0??0001?0?0???[01]?00??00???????????0???1??0????????0????????????2??????0??000???0???0?????????????01?0?00???0?1??????????1?1???1?0?00000??0??0??00001??0????0??????????????????0??????0?0???0?????0???????0?0?00??00???0??1???1?0???????????????[01]?0000?01[01]00?000???0??0????????????0?0????11??0??????01?????????000??

MPC-D 100/140 ?????????????????????????????????????????????????????????????????????????????????????????????????????????????????????????????20??1????????????????????1?0100100000000000???????????????????????????????????????????????[01]00[12]10??101?????????????????????????????01000??????101??021????10???10????????????????????2?????????????????????????????????????????????????????????????1?1???2001112?????11??003?0??0??10?????????0??????????1001?0??????1???????????????????????????????000????????????????????????0????????????????????0?????1?11?0??????????1???????????????????????????????????????????????????????????????????????????????????????????????[01]?0000?0?[01]0??????????????????????????????011?00???1?????????00???0???

MPC-D 100/972 & 100/974 ?002100????1?0001?111111011010010???1???0102021?00??????????1?1001100000010000??1????0?0?11??0020?????????????????????????????????????????????????????????????????????????????????????????????????????????????????????????????????103??10?0?????1????????????????????????????????????????????0???0????????0?11???????000011?????000001010?1?0????01000210[01]1???10??0???????????????????????????????????????????0????0?0???0???0???????????????????????0?0??0?0?????????????????????????????????????????????1??0?0???0?0??????????1????11??????10??0???????00???000??0001?0???0??????1??0???????????????????????????????????????????????????????????????????????????????????????????????????????????????110????????????????0??

*Almas* ?002010010?0??11?????011???1100??11?1???01?202??0?1?1001????1??0??0???0?00??0??2??0?000021[12]??0020????????????????????????[01]?1?[12]??1????1??????????????????????????????????11[12]????[01]???01?20002[12]1[12]?[12]001???[12]1?1010?00??1?01?0??20?????21030?100?1????[01]??????[12]??????0?????0??00?????0???????????1??0?1?0?0???????0111???010010?11??1?100000100?010????1010002000101?0??00???0?????????????????????1?11011????3??????01???00???0????00?????????????0?????00?1?01?1???????????????????????????????0?00??????0???0?10??10???0?0??????????0????????????00??01??0??001???000????0?0?0??00??????0???????0????????????0?????????????????????????????????????????????????????????00??????0?00100?????01?0??00????????1???10????????????00?

*Jinfengopteryx* ?0?3?0001??????????????[12]??????0?????????0??102100?001????1??????0?????0??101?0??1?10?000211??[01]020??1??????????0??????????1??0212110001????111011?200?[01]???10000000000[01]000?1[12]??1??????0?2?0???11???10????1???0???????????0??1?0??001002??00???11??0?020??2?001?2011?0?0?101???????[12]?110?1011?[01]?0???010????????0?10???1?00?0121?????001000??01?1101101000?000100??0?00?[012]?1?21?0011???0002[01]000121?1?0?1???0[23]?????0?1?1?0?????000??????????????1????0????1?????0?1?????????01?00???1???00???????1?????????0????1????0???00????????0????????1?01???0?0?0?????000?100?00??0?????0???????????????????0?01??0?0?1?0???????????000?0??00010?????00???????????????[01]?0000??0[01]0000?????0??0???????????1???????????0??????0?0???0????0????

*Liaoningvenator* ??0200101?0????1???0000??11[12]0?01010?1?00???2?[12]0?00011??10??110????0??000000010?[12]0??????0112100020??00111??00001??0?0?????1??020???00??????111011121010????????0000?0[01]1??010211????[01]?121?0?[123][12]11???1??01100??0???0????1?00??100????11?2?0?01?10???0?03[01]?02??0001?01[01]??0???0?111000????????0????0?1001?0?0???0001?11[12]010???011?0?[12]1000?0?0???1??1??1011002?0110?1?0??0211??2111010???00?20000?[12]1?1?0111?003100?000??1?00??1??00?100??1?????????0?????0???????0???????001?01?110??0???0010?[01]?????????????10?0?200?00????0??????0?0???02?101??100??00101?00?00?01??000?0??0???0??????????????????100?1??000?0?0????????????1??0????01010????0???0?????0?????0??0??????000?????00?0???1?1???????0??00??[01]1??????0?10??????10??001??

MPC-D 100/44 ???????????0?01?0?1?????0???????????????????????????????0?01?0??????????????10?11???????1??0????????010???0??????????????????????????????????????????????10000[012]00000[01]000???????????????????????????????????????????????[01]01[123]00[12]0001????????????0?[01]?????????????0?1?????????110???[01]??????0??000????0?0?100??0?00???2??????[012]??1?????????????????????????????????10????????????????101000?00?00??????11?0?1[23]?0??0???0?????0???0?0????????????????????0?????0????0????????????????????010??????????????????????????????????1???1??????0????11??100???????????????????????????????????????01???????????????????????????????????????????????????????????????????0000000[01]0???????????????????????????????11?0????0??????????0???0???

*Daliansaurus* ?0?[123]??10?0??????????????????1?010??0?????????????????????????????????????0001???1??0?000111000020?1???11??100?11??000???01???2011?01?????????????20??0????0??[01]0000?0100?0111011?1???0?2?[01]???1???????2?211?????10????0??00??0020??2103???0???????0?0?1?01?????1?010??00????1100?02??????01??[01]???110?0??0????001?011?0?????111??21?0????0??0???1??10????2?00????0??102??0??????111?100020011121?1?0110?0[01]3?0???0010??????1??00??????1?????????0?????0??????????????0001??2??10?00??10010???0????????????0???100????0??0?????????????????00?1?00?0[01]?0??00?????1???00?????????????????????0?????0???00??0??0?????????????????????????1?????0??0??????????[01]???0000001[01]??0???0?010???1?[01]??????????[01]????01??0???0?????????1????0???

*Tochisaurus* ??????????????????????????????????????????????????????????????????????????????????????????????????????????????????????????????????????????????????????????????????????????????????????????????????????????????????????0001200[01]???2??????????????????????????????????????????0?????????????????????????0?????????????0????????????????????????????????????????????????????????????????????????????111???3?????????????????????????????????????????????????????????????????????????????????????????????????????????????????????????0????????10?????????????????????????????????????????????????????????????????????????????????????????????????????????????????????????????????????????????????00?001?????????????????1???????

*Hesperornithoides* ???3111????????????????????????10???01??0110???????????????1???????????0?[01]0?10?????????0[01]10100???1??1?01??000???1[12]1??????????11??10??????0???????2[01]0000??10000[01]000001000?????1?1???????????????????????1?0001100001?0000??100[12]0?01??????[01]???????[01]1???????????[12]00?????????1???0???1????10???0?0???0?0????1?0?00?11???0???2?11???1100001?1?????1??1?1100[12]?00????00???2???0?????1010??00????10[12]??11[01]11?0?1[23]?01?0?01??10???1?00??????1???00021?????0??000?1?????00????00?102001????10000??????????????0000000?110??????0?0???00?????0????11??????00?10?000?0?0????????????????????????0?0???????0?0???10?????????????000????0???01???1?1?1000?00?0?1???????0??00001???????????????000[01]100200000?00000?1??0???????0???101????????

*Sinusonasus* ?0030110???????????????????1100?????????01021?????????????????????????0?000?0????????0001[123]11000?????????????????????0????21??21101?????????????????????????????????????????????????0[01]02?00?[12]1????0??2??????0??0???11??10??1?02010?001??11???????[01]?????01????????????????0?111?00[01]1????0??????0???0????0?????0????210?11??11?????0000000???????0?2010002000????????????0???????????????????????1?011?01?3?0??0?01???0????0?????????????????????????0???????0?0?????????????????????????????0?0????0???00????00??????0????????????0??????0???0?00??0??????00????000??????????????????????????????????????0?0????????????????????????????????????????????????????????000??????????????[01]??????0????00????0???0???????????????0??

*Xixiasaurus* ?003011????????????????????111010???????00021???????????????????????0?1??[12]??0????????0002[123]1??00?0?1???????????????????????????????????????????????????????????[0123]00??0?0????????????????????????????????????????????????????????????0010000???????[01]??????????????0????????????????????????1????1???[01]????????0?01???????100?11?????000[01]010??0???????01100?0?01???0??10???????????0???00??0?01??????????????????????0??0??????????????????????????????????????0?0?????????????????????????????????????????????[12]?????10?0?0??????????0????????????00?10??????????00100??00????????????????????????????????????????????????????????????????????????????????????00????0????????????????????????????????????????????????????????00??

*Byronosaurus* ?0030??????1?01?0??????1111010010???????010?1?0?????????????1?100?????0000001????????000231??002?10?0?01?110001010????[01]??????20???????????????????????????????????????????????????????????????????????????0?[12]?0?????????0??????????02??11?????1?0????????????????????????0?0?????????????????0???0???1?0??0?0????2????1?111?????00000001?0??0????012002?00???100?102?????????????????????????????????????0????0????0???0????0?????????????????????00?1?0??100?????????????????????????????????????00??????[01]??0??00?0????????????0????01??????10??0??0????00?00000??0???????????????1??00??????0??010????????????????????????????????????????????????????????????????????????????1?????????????????????1101???????????????0??

*Sinornithoides* ?003011010?1???00??1??1????0?001?00?1?0001?[12]?2??0?1??00101????????????00010?10?21?0000001[123]101002????0?011??0001??[12]??1??0?01?0211111011????1?00110100000??1000000000010102122?1011?2010200?211112110?2?210??0?000???1010001[12]00201011020100???????0?1???1[12]??0011?0100010000?011[01]00[12]1???01011?[01]00?1101?0?0?000001?00210?0??0011?2210000000??010???120110?2000001?00?002??00111?0111110002001012??1?01110003001000010010???[01]000????00?0010?0?10?0??0??000?????0000?????????0100?0?0???10?0?2000?00??1?000?0?00200??0??0??????????????????10101?00?0?101?00?00??10000?????????????0?????1???0????000??0?0???0?0????????1??000?01?010?01??1100??0010?1?????[01]?0?0000??000000??00??000????000[12]000?000000011?00?1?111??00??0??0?0011?

IVPPV11119 ???????????????????????????????????????????????????????????????????????????????????????0[01]?????????????????????????????????????????????????????????????????????????????????????????????????????????????????????????????0[01]00[123]?????01????????????????????????????????????????10??????????????????????????0?????0????????????????????????????????????????????????????????????????????????????????????11????[23]?0??????????????????????????????????????????????????0????????????????????????????????????????????????????????????????????0???????????????????????????????????????????????????????????????????????????????????????????????????????????????????????????????????????????????????????????????1??????????????????0???????

*Gobivenator* ?0?301001000?1100?11101000???00100001100010212100?11100100011110010000?0000010?1110000?02[23]1??0??01??01111110001011?11100111002011100??????011010120000?0????????????????[01]??10110111011102021110100001111000?11000010010001210[23]0??20?[23]??1?0011??10?120[01]01??000111????0?0000011?00?????11??10???0110100100000?01?0??0000?00?1?1221000001010?1001?110110020001011000??2110?21?101??????????????1??001110??32?100?01?110000[01]00??0000??00????????0???0?0?0??0?0??0000?0001?0000000?000????0?1100?010?11???00??0[01]?0?10??0000?1???1?00000[01]1[01]?1???10?000?01?00??010?0??001000010?0???00??0?10??????100001010?000?0???????????00??00?0101?1?0?100??00?001???0??????????????000??01??0??000?00010???00?000?01??00??1?00??????00100?000

*Urbacodon*_holotype_only ??????????????????????????????????????????????????????????????????????1?000?1????????????3???0020???????????????????????????????????????????????????????????????????????????????????????????????????????????????????????????????????????????????1????????????????????????????????????????????0???0??????????01???????????1????????????????????????????????????0???0???????????????????????????????????????????????????????????????????????????????????????0?0?????????????????????????????????????????????????????????????????????????????????0??????????????00????????????????????????0????????????????????????????????????????????????????????????????????????????????????????????????????????????????????????????????????

*Geminiraptor* ???3001????????????????????????1???????????????????????????????????????????????????????0????????0?????????????????????????????????????????????????????????????????????????????????????????????????????????????????????????????????????1??????????????????????????????????????????????????????????????????????0???????0??????????00000[12]0???????????11?????0???????????????????????????????????????????????????????????????????????????????????????????????????????????????????????????????????????????????????????????????????????????????????0??10???????????????????????????????????????????????????????????????????????????????????????????????????????????????????????????????????????????????????????????????????????0??

*Borogovia* ?????????????????????????????????????????????????????????????????????????????????????????????????????????????????????????????????????????????????????????????????????????????????????????????????????????????1?0101101?[01]00[123]0???102????????????????????????????????????????0111??[01]0????????????????????0??????????2?????????????1?????????????????????????????????????????????????????????????????11????[23]????????????????????????????????????????????0???????????????????????????????????????????????0?0?0??0?????????????????????1?????1?1101?????????????????????????????????????????????????????????????????????????????????????????????????????????????????????????????????????10010000???????11??????1???????????10????0

*Saurornithoides* ?0031000??01???00?111011?11011010???1??????[12]?????????????1?0???0???00110?00?101?1????000131000020??????????????01[01]??1100??1????????????????????????????????????????????????????????0101000[23]011??11[01]?11110??????????????[01]?0[123]00[23]0?020020?10?????1?1??1?01?????????????????0?01???0?????????????0??10????00?00?01???[12]10?10?0?1??22?00000?01?0???????011002000?0?1?0?10??10???????????????????????1??11?0??[23]00??0?0????000??00??0????????????????????????1???0??0?????????????????????????[01][12]??0?0??01????????12??0??00?0?0??0???????002???10???0??0??0??????010?00?00?0000??00????????0?0?00?????????010??00??????????????????????????????????????????????????????????000????????0???????????????????0[01]???0101??????????0????0??

*Zanabazar* ?003000010?1?1100?11101001101101?01?01??010212000?0110??????111001????1?000?1???1????000131000020?1?????????????????11100010?21?11?????????????????????????????????????????????????????????????????????????0?0?010110110??2???????0020?000000?111????001??????????????????????????????1??????00??0???1?1??0?01???????00??11????100000001?01?0????01100200010?000?10???0????????????????????????????1??????????01??10????0???000??????????????????????11010000???????1???????????????????????????????????0?20011000?0?0?1??????0?0????1???????00??0???0???1??00000????01[12]????00?????1??00???100????????00??????????????????????????????????????????????????????????????????????????1011000??00????????0010???1??????0?00??0?0

*Troodon*_sensu_lato ???03?0001??1?1100?1110100110???10?0?????0002120[01]0001101?0??11[01]0001??0?1?0[01]001????????0?0[01]3101002011???01111[01]001011111[12]00??10021??11???????1110?1120?1001?100?1[012]0???0[01]???011211?01100001001?111011[01]?011[12]1000[01]10001?1[01]0[01]100[01]20020102?0?????1[01]00?1110?1?0[01][12]??0??110?????[01]000?0101?010???000???00[01]?1[01]0??0101?000010[01]021000???01?12?10?00000???1?0[01]??[01]0?1?????010?100??02[01]1??21?101110?????[01]?????1?1?011100?210200??1?01????[01]0??10000??011??0[12]1??0??????0[01]1100?0100??????1??2?010?1???0??1011100?0?0?1???000?0??0?11000??0??1????000??0[12]????1?1000?00????00??0????0[01]0?????01[12]????00????01??0?????0000?010???0?0????????????0?????0?0[01]?????0?0???0??0??000001??0?00?1???[01]00??010?0?0????10010001??0??100[01]???01011?1???????[01]00???01

*Talos* ?????????????????????????????????????????????????????????????????????????????????????????????????1?????????????1?[01]????[01]??????2???????????????????????01????????????????????????????1[01]01?00[12]?????11[01]???11???1[01]1?01011001000[12]1020002?????????????????11??[12]???????????????00?1111?0[01]???????????????1?????0??????????2100?????????21????????????????????????????????????01????????1??????????????????11100?3?0??0?????1???????????????1?10?0[12]?????????????????????00?000????????????????????????????????00??0??0????????????0????????0?????1?1100??????????00????????????????????????????????????????010????????????????????????????????????????????????0?1?????????????0?0??????0????1??00??1??0??1001??????1??????????10?????0

*Tamarro* ???????????????????????????????????????????????????????????????????????????????????????????????????????????????????????????????????????????????????????????????????????????????????????????????????????????????????????[01]0?2?????????????????????????????????????????????????????????????????????????????????????????0????????????????????????????????????????????????????????????????????????????11??????????????????????????????????????????????????????????????????????????????????????????????????????????????????????????????0????????1????????????????????????????????????????????????????????????????????????????????????????????????????????????????????????????????????????????????00???0???????????????????????????

*Philovenator* ???????????????????????????????????????????????????????????????????????????????????????????????????????????????????????????????????????????????????????????????????????????????????????????????????0[12]01101?1?1?01010111001200201?2???????????????????????????????????????01110000???????????????1?????0??????????2??0?????????21??????????????????????????????????????????????????????????????10011100?3?0??0??????????????????????????????????10?00??1???????????????????????????????????????0010?00???00?0?????????????????????0?????1?1000?????????????????????????????????????????????????????????????????????????????????????????????????????????????????????????????????0?0?00000000010000011??????1??????????000????0

*Linhevenator* ????0??0???????????????????????1?0?0???000?21??10??1101?????0?00??1????0?????????????0?01?[01]000????1????????????????????????????????????????0?0??02101????????????????????????????????1[12]?0?????????????10?10????????????0??20020102????????01??????01?????????10?????????0?1111?0[12]0??????????????1???0?0???0?0????2100????0???????0?000?1???0????1???002?0?10?1??1??????????0?1??????????????????011011?[23]?0[12]00?1????00??????????0????????????????????????1???0?0?0?????0????0??100??????????????????????????????010?0?????????????0???????1?0?10??01?????01?????00????????????0??????????????00?????1???????????????????????????0??????00???????1?00??????????????????????????????????????????00100[01]??????1??1???????1????0??

*Hypnovenator* ?????????????????????????????????????????????????????????????????????????????????????????????????????????????????????????????????????????????????????010010000000?00?0?0?????????????????????????????????1001100001001?0??20?????2????????????????????????????011010?????0??11??10?????00??00???????????0???????????0??????1???1?????????????????????????????????????????????1111100020010?2???0?111???3?010???10?1???????0???????101?101????????0100?1????????????????????00?00000???????????????00?0?00??00????????????????????????????????????????0?0???[012]?????????????????????????????????????????????0?????????????????????????????????????1?000?0?0?000000000????????????001?0001000000000????????????????????0?00?0??0

References for Supplementary Data S2

1 Sellés, A. G. *et al*. A fast-growing basal troodontid (Dinosauria: Theropoda) from the latest Cretaceous of Europe. *Sci. Rep.* **11**(1), 4855; [10.1038/s41598-021-83745-5](https://doi.org/10.1038/s41598-021-83745-5) (2021).

**Supplementary Figure**


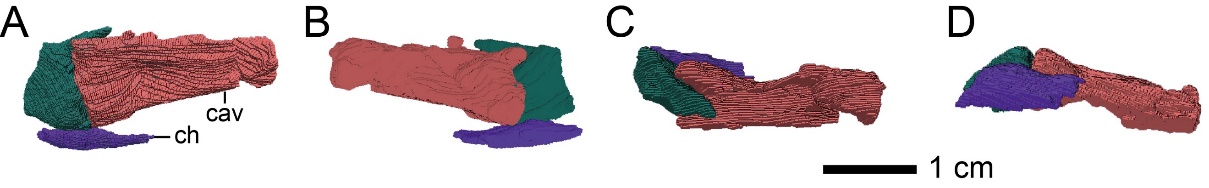


Supplementary Figure S1. Distal caudal vertebrae and a chevron of *Hypnovenator* *matsubaraetoheorum* gen. et sp. nov. in left lateral (A), right lateral (B), dorsal (C), and ventral (D) views. Abbreviations: cav, caudal vertebra; ch, chevron.


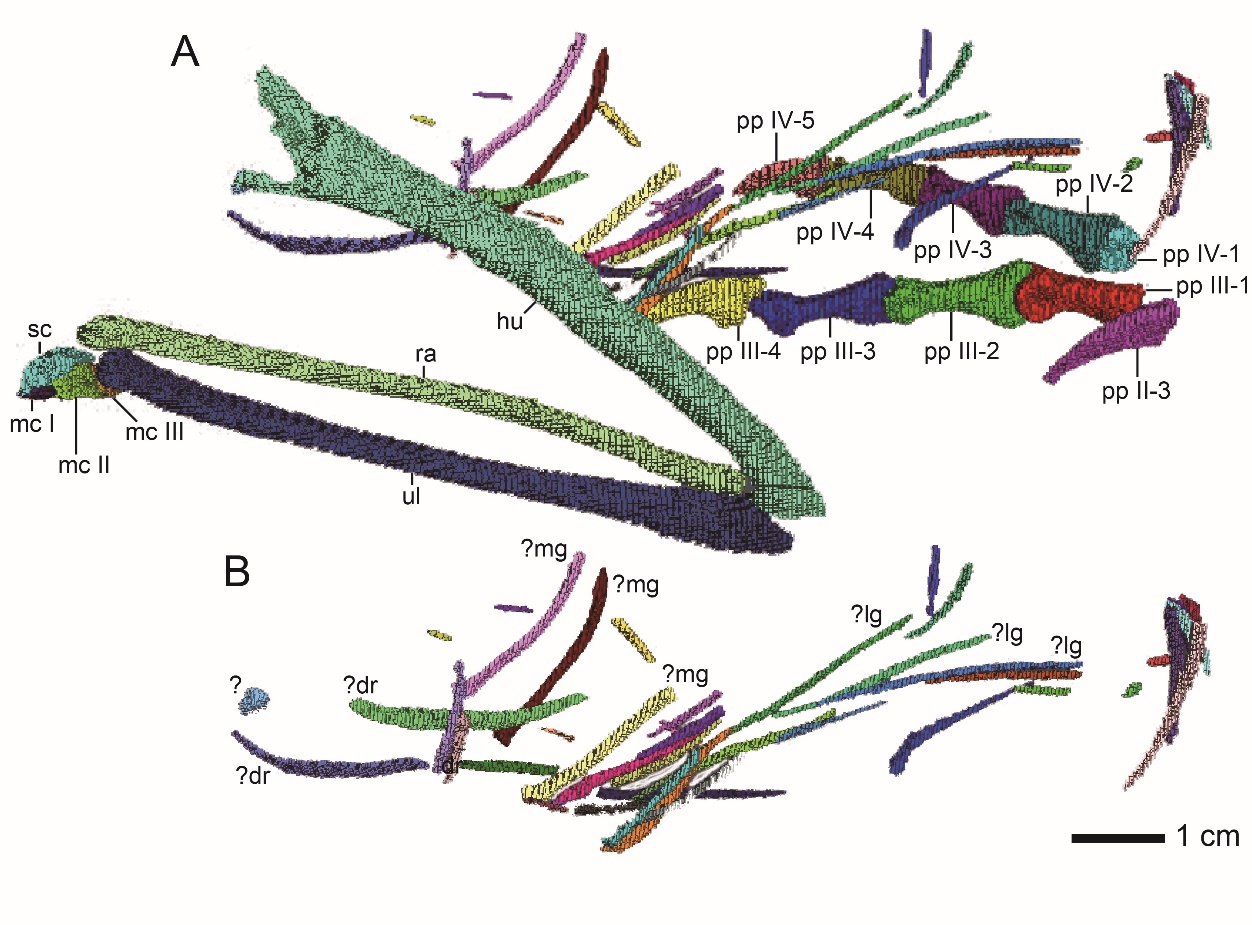


Supplementary Figure S2. Arrangement of left arm, left wrist, gastralia, and right pes of *Hypnovenator* *matsubaraetoheorum* gen. et sp. nov. in left lateral view (A) and arrangement of gastralia (B). Abbreviations: dr, dorsal rib; hu, humerus; lg, lateral gastralia segment; mc I, metacarpal I; mc II, metacarpal II; mc III, metacarpal III; mg, medial gastralia segment; pp II-3, pedal phalanx II-3; pp III-1, pedal phalanx III-1; pp III-2, pedal phalanx III-2; pp III-3, pedal phalanx III-3; pp III-4, pedal phalanx III-4; pp IV-1, pedal phalanx IV-1; pp IV-2, pedal phalanx IV-2; pp IV-3, pedal phalanx IV-3; pp IV-4, pedal phalanx IV-4; pp IV-5, pedal phalanx IV-5; ra, radius; sc, semilunate carpal; ul, ulna.


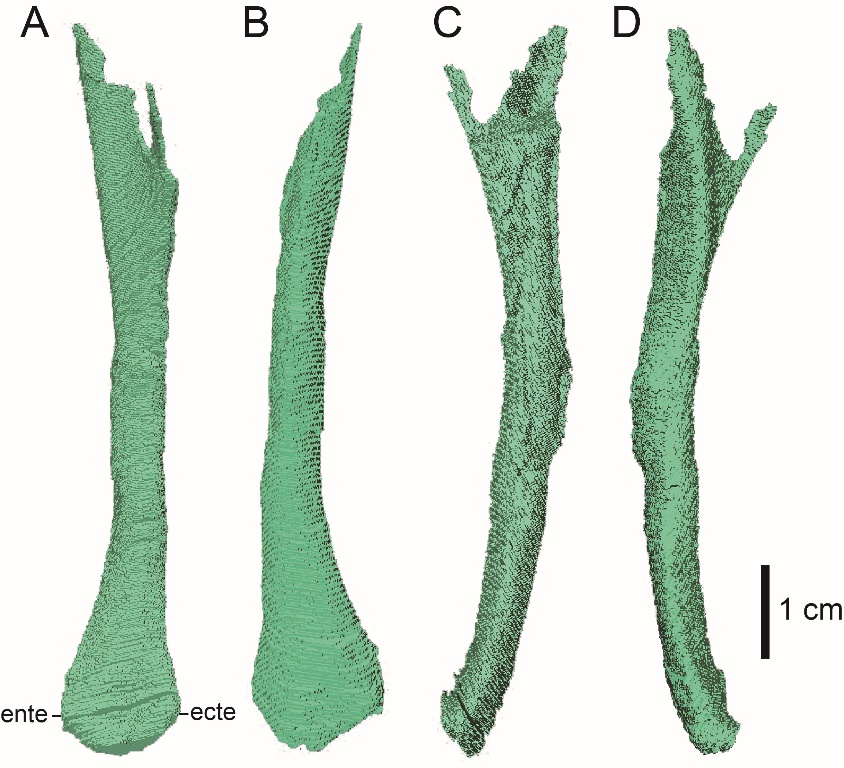


Supplementary Figure S3. Left humerus of *Hypnovenator* *matsubaraetoheorum* gen. et sp. nov. in anterior (A), posterior (B), lateral (C), and medial (D) views. Abbreviations: ente, entepicondyle; ecte, ectepicondyle.


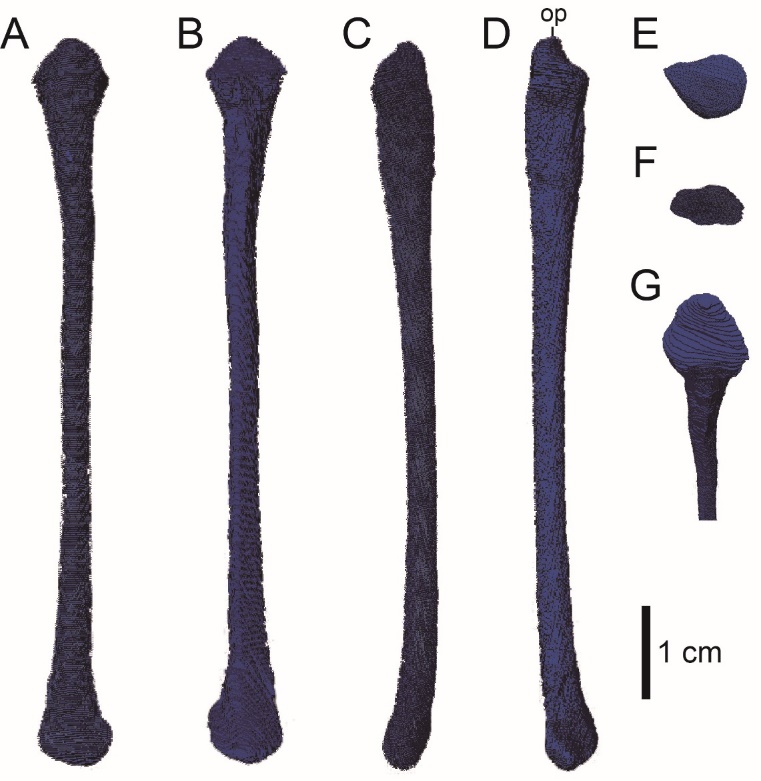


Supplementary Figure S4. Left ulna of *Hypnovenator* *matsubaraetoheorum* gen. et sp. nov. in dorsal (A), ventral (B), lateral (C), medial (D), proximal (E), distal (F), and proximodorsal views (G). Abbreviations: op, olecranon process.


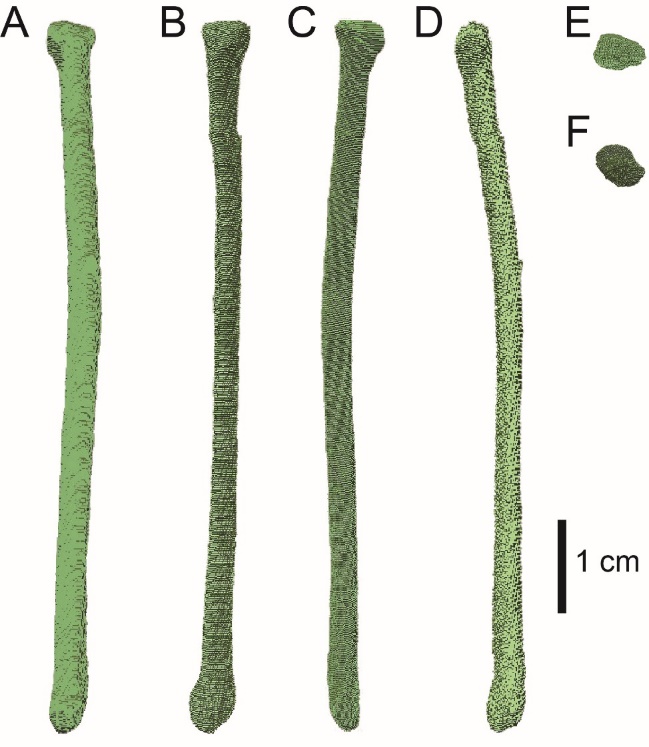


Supplementary Figure S5. Left radius of *Hypnovenator* *matsubaraetoheorum* gen. et sp. nov. in dorsal (A), ventral (B), lateral (C), medial (D), proximal (E), and distal (F) views.


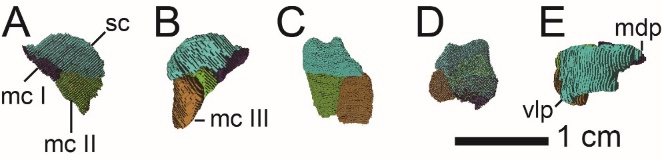


Supplementary Figure S6. Left distal carpal and proximal ends of metacarpals I to III of *Hypnovenator* *matsubaraetoheorum* gen. et sp. nov. in dorsal (A), ventral (B), lateral (C), medial (D), and proximal (E) views. Abbreviations: mc I, metacarpal I; mc II, metacarpal II; mc III, metacarpal III; mdp, mediodorsal process; sc, semilunate carpal; vlp, ventrolateral process.


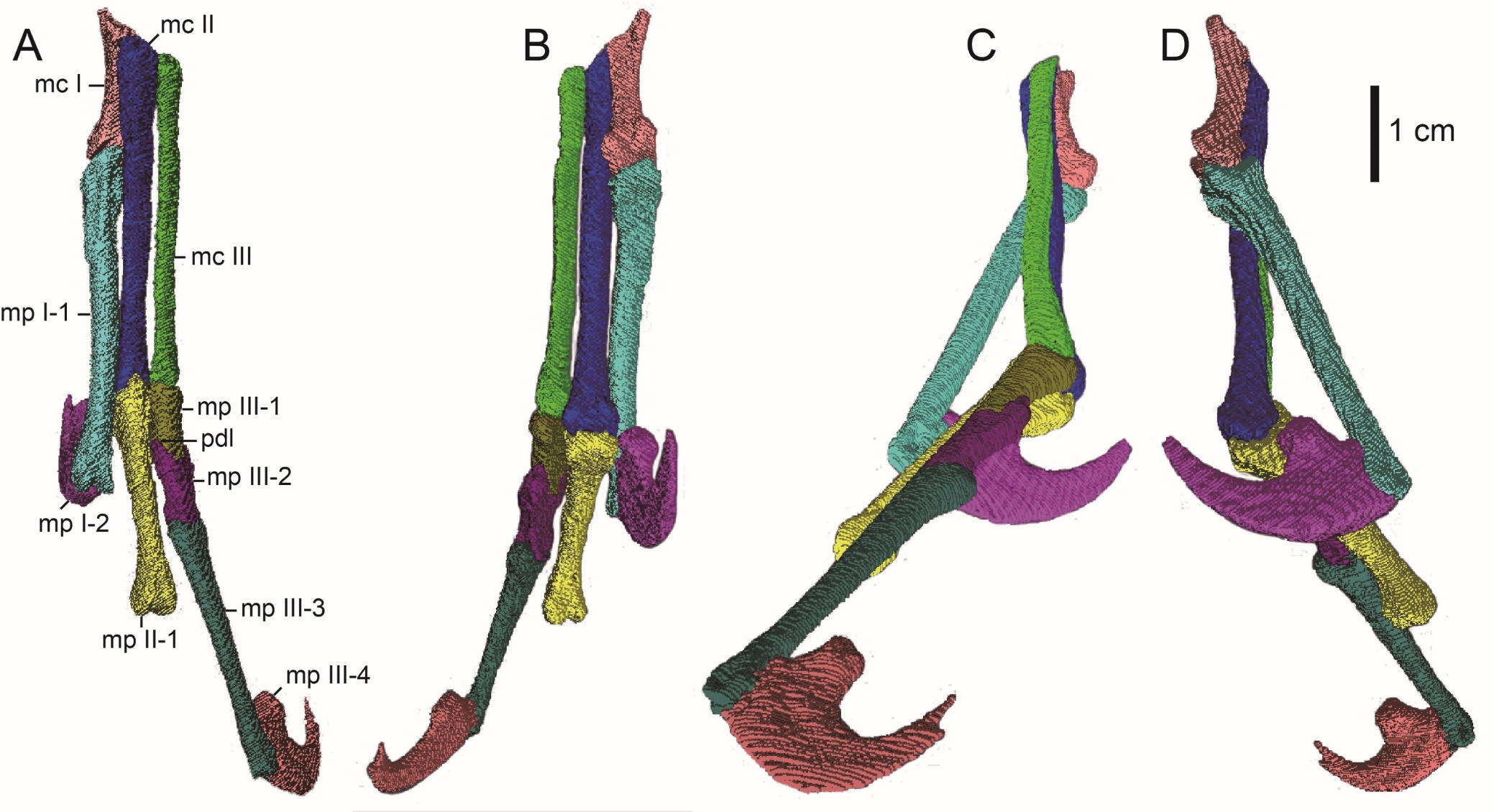


Supplementary Figure S7. Left manus, missing the proximal ends of metatarsals I to III and manual phalanges II-2 to 3, of *Hypnovenator* *matsubaraetoheorum* gen. et sp. nov. in dorsal (A), ventral (B), lateral (C), and medial (D) views. Abbreviations: mc I, metacarpal I; mc II, metacarpal II; mc III, metacarpal III; mp I-1, manual phalanx I-1; mp I-2, manual phalanx I-2 (manual ungual phalanx I); mp II-1, manual phalanx II-1; mp III-1, manual phalanx III-1; mp III-2, manual phalanx III-2; mp III-3, manual phalanx III-3; mp III-4; manual phalanx III-4 (manual ungual phalanx III); pdl, proximodorsal lip.


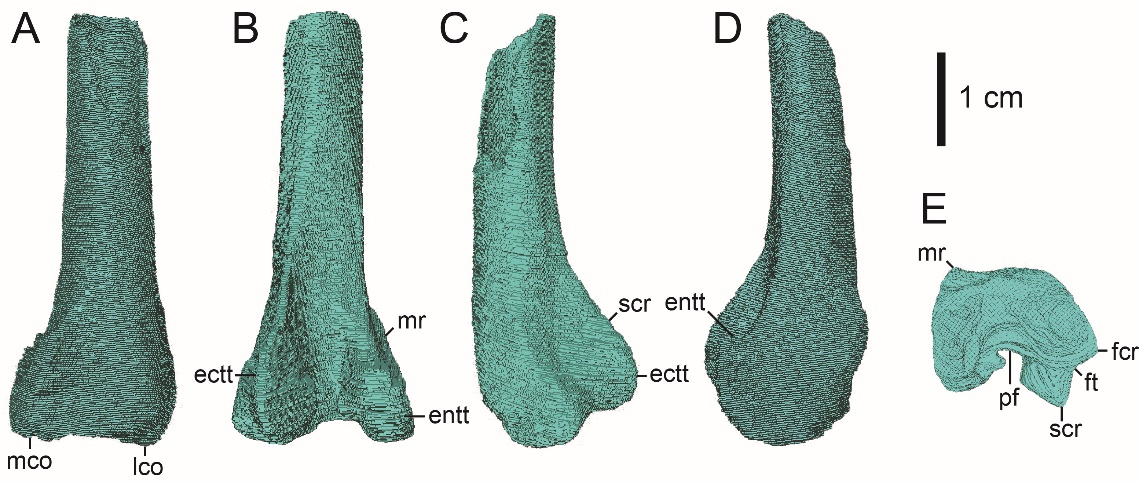


Supplementary Figure S8. Left femur (distal part) of *Hypnovenator* *matsubaraetoheorum* gen. et sp. nov. in anterior (A), posterior (B), lateral (C), medial (D), and distal (E) views. Abbreviations: ectt, ectocondylar tubercle; entt, entocondylar tubercle; fcr, fibular crest; ft, fibular trochlea (= trochlea fibularis); lco, lateral condyle; mco, medial condyle; mr, medial ridge; pf, popliteal fossa; scr, supracondylar crest (= lateral posterior ridge, tibiofibular crest).


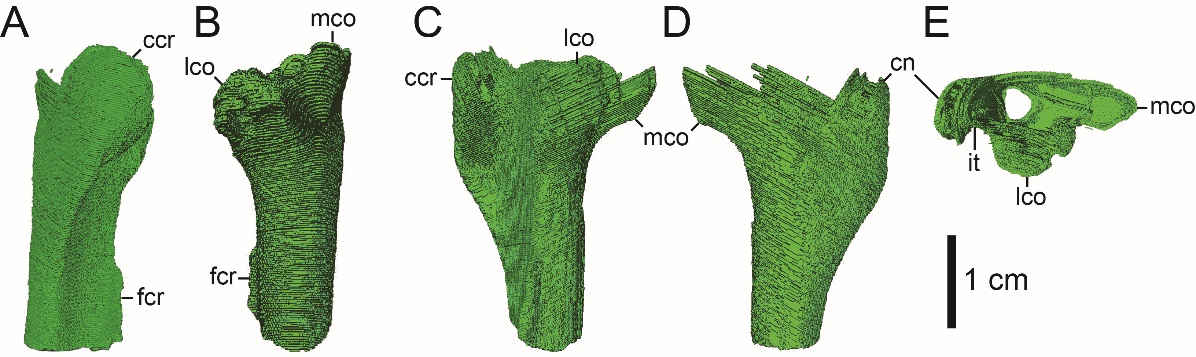


Supplementary Figure S9. Left tibia (proximal part) of *Hypnovenator* *matsubaraetoheorum* gen. et sp. nov. in anterior (A), posterior (B), lateral (C), and medial (D) views. Abbreviations: ccr, cnemial crest; fcr, fibular crest; it, incisura tibialis; lco, lateral condyle; mco, medial condyle.


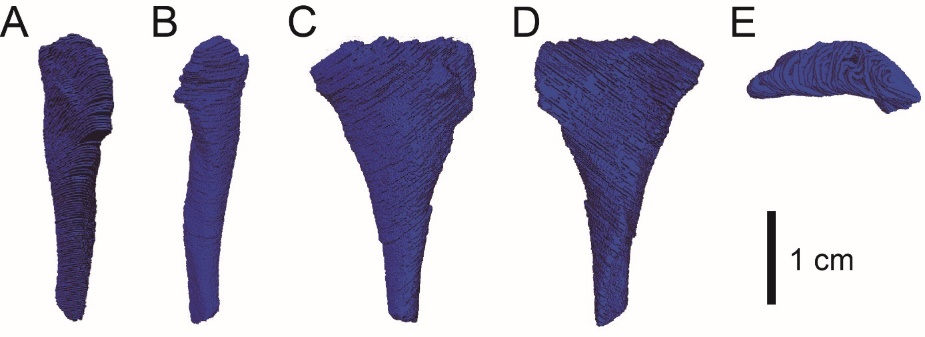


Supplementary Figure S10. Left fibula (proximal part) of *Hypnovenator* *matsubaraetoheorum* gen. et sp. nov. in anterior (A), posterior (B), lateral (C), medial (D), and proximal (E) views.


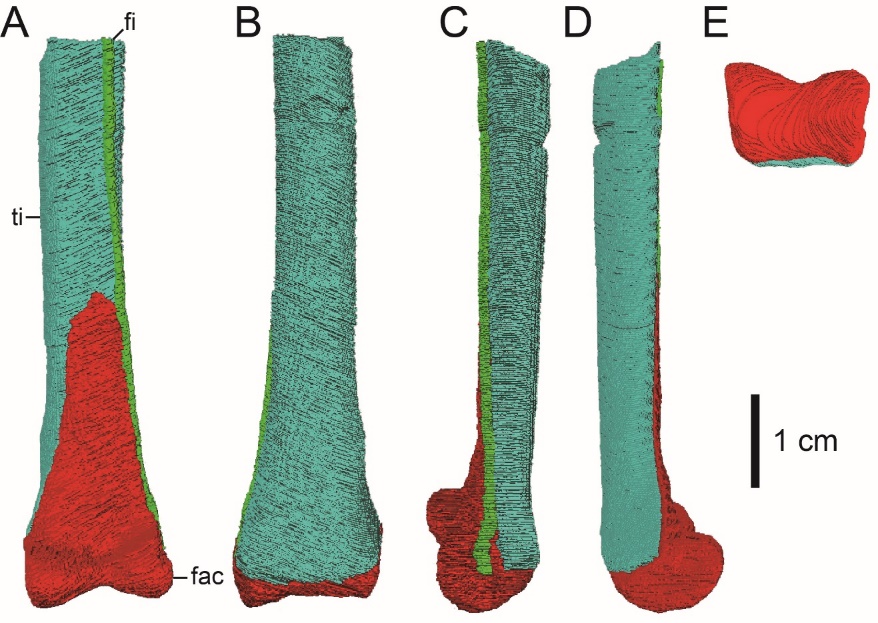


Supplementary Figure S11. Left tibia (distal part), fibula (distal part), and fused astragalus and calcaneum of *Hypnovenator* *matsubaraetoheorum* gen. et sp. nov. in anterior (A), posterior (B), lateral (C), medial (D), and distal (E) views. Abbreviations: fac, fused astragalus and calcaneum; fi, fibula; ti, tibia.


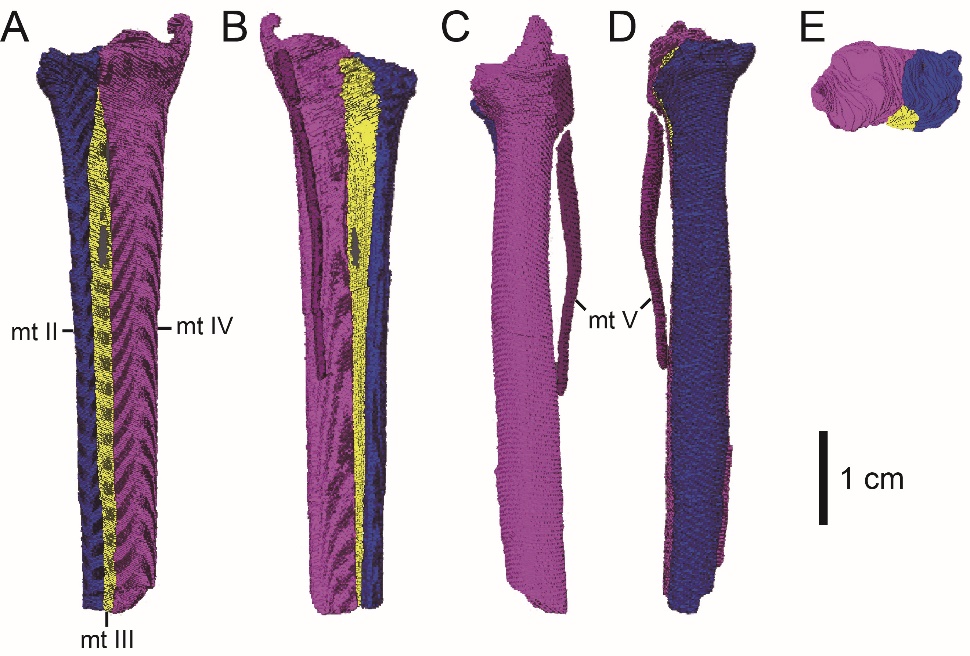


Supplementary Figure S12. Left metatarsals II to V of *Hypnovenator* *matsubaraetoheorum* gen. et sp. nov. in anterior (A), posterior (B), lateral (C), medial (D), and proximal (E) views. Abbreviations: mt II, metatarsal II; mt III, metatarsal III; mt IV, metatarsal IV; mt V, metatarsal V.


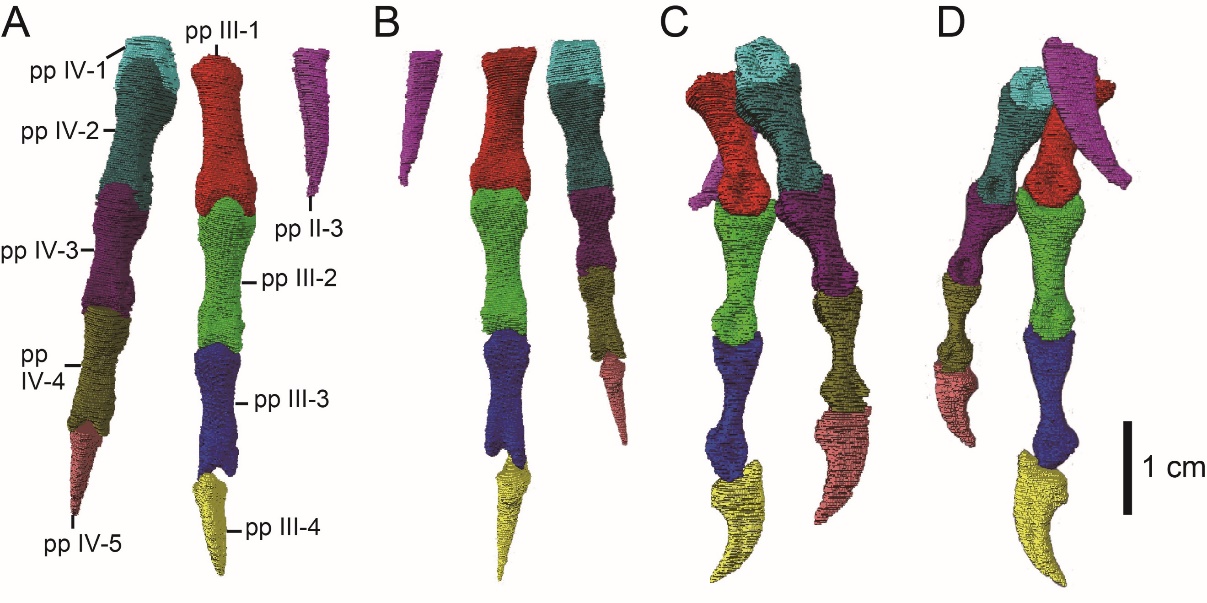


Supplementary Figure S13. Right pedal phalanges II-3, III-1 to 4, and IV-1 to 5 of *Hypnovenator* *matsubaraetoheorum* gen. et sp. nov. in dorsal (A), ventral (B), lateral (C), and medial (D) views. Abbreviations: pp II-3; pedal phalanx II-3 (pedal ungual phalanx II); pp III-1; pedal phalanx III-1; pp III-2; pedal phalanx III-2; pp III-3; pedal phalanx III-3; pp III-4; pedal phalanx III-4 (pedal ungual phalanx III); pp IV-1; pedal phalanx IV-1; pp IV-2; pedal phalanx IV-2; pp IV-3; pedal phalanx IV-3; pp IV-4; pedal phalanx IV-4; pp IV-5; pedal phalanx IV-5 (pedal ungual phalanx IV).


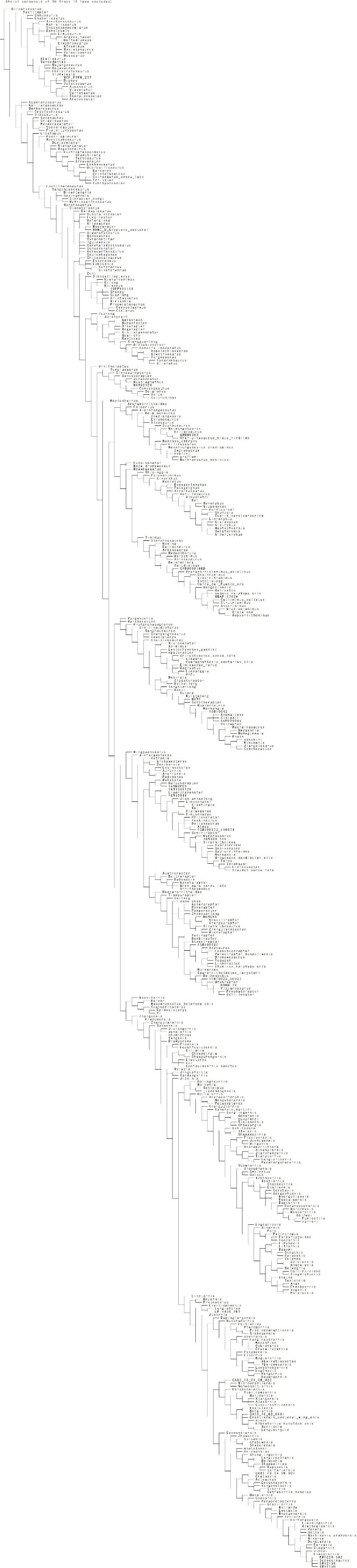


Supplementary Figure S14. Phylogenetic analysis. Strict consensus tree of 10 MPTs in this study.


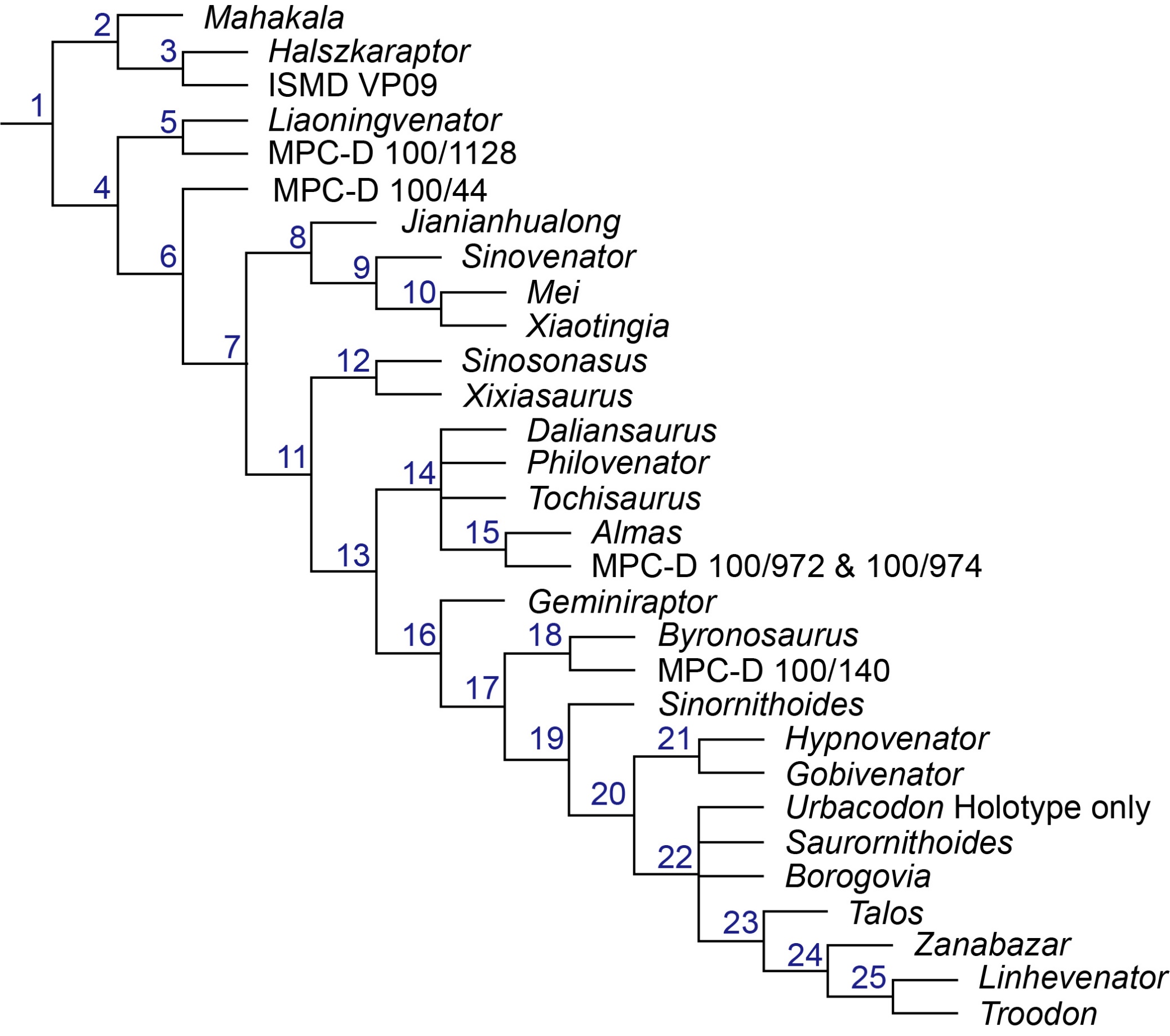


Supplementary Figure S15. A strict consensus tree of 10 MPTs obtained from the phylogenetic analysis in this study. The blue numbers above each branch show node numbers.
